# Urinary carbonic anhydrase 1 excretion is a marker of hemolysis-triggering conditions suitable for point-of-care testing

**DOI:** 10.1016/j.bglo.2025.100058

**Published:** 2026-03

**Authors:** Alzbeta Hulikova, Zhenyi Wang, Helen Broomfield, Joanna Robinson, Miryam Healy, Rómulo J. Figueroa-Mujíca, Dionicia Gamboa, Katherine Torres, Gopal Ammanath, Deny Hartono, Richard C. Siow, Sara Engledow, Ketsanee Srinamon, Aniruddha Ghose, Katherine Plewes, Arjen M. Dondorp, Noemi B. A. Roy, Francisco C. Villafuerte, Hammad Khan, Pawel Swietach

**Affiliations:** 1 Department of Physiology, Anatomy, and Genetics, University of Oxford, Oxford, United Kingdom; 2 Neonatal Unit, Evelina London Children’s Hospital, St. Thomas’ Hospital, London, United Kingdom; 3 Laboratorio de Fisiología del Transporte de Oxígeno y Adaptación a la Altura-Laboratorios de Investigación y Desarrollo, Facultad de Ciencias e Ingeniería, Universidad Peruana Cayetano Heredia, Lima, Perú; 4 Laboratorio de Malaria-Laboratorios de Investigación y Desarrollo, Facultad de Ciencias e Ingeniería, Universidad Peruana Cayetano Heredia, Lima, Perú; 5 Camtech Diagnostics Pte Ltd, Singapore; 6 School of Cardiovascular and Metabolic Medicine & Sciences, King’s College London, London, United Kingdom; 7 Department of Haematology, Oxford University Hospitals NHS Trust, Oxford, United Kingdom; 8 Mahidol Oxford Tropical Medicine Research Unit, Faculty of Tropical Medicine, Mahidol University, Bangkok, Thailand; 9 Department of Medicine, Chattogram Medical College Hospital, Chattogram, Bangladesh; 10 Centre for Tropical Medicine and Global Health, Nuffield Department of Medicine, University of Oxford, Oxford, United Kingdom

## Abstract

Intravascular hemolysis accompanies diverse diseases, yet blood-based markers (eg, plasma-free hemoglobin [PFH], haptoglobin, lactate dehydrogenase [LDH], and bilirubin) are variably sensitive, nonspecific, or impractical for point-of-care use. We evaluated urinary carbonic anhydrase 1 (CA1) as a mechanistically grounded, urine-based marker of hemolysis in a multicenter study spanning the United Kingdom, Bangladesh, and Peru. We enrolled 234 participants: healthy adults and adults with inherited anemias (Oxford), newborns in intensive care (London), children and adults with complicated or uncomplicated malaria (Bangladesh), and adults attending rural clinics for various medical reasons (Peru). Urine CA1 and hemoglobin (Hb) were quantified by enzyme-linked immunosorbent assay and immunoblot, with CA1:Hb stoichiometry used to distinguish intravascular hemolysis from urogenital blood contamination. Relationships with PFH, LDH, bilirubin, Hb, C-reactive protein (CRP), and clinical variables were tested using regression, principal component analysis, and decision tree. Reference urine samples were predominantly CA1-negative/Hb-negative. In inherited anemias, urinary CA1 was highest in sickle cell disease and correlated with serum LDH and inversely with blood Hb. In neonates, longitudinal CA1 trajectories stratified infants into physiological, transient, and sustained hemolysis groups; higher CA1 levels were associated with prematurity, elevated CRP and lower white blood cell count. Urinary CA1 was detected in Bangladeshi patients with elevated PFH and in cases of intravascular hemolysis not identified by PFH. Among Peruvian participants, urinary CA1 correlated with raised CRP level, consistent with inflammation being a prohemolytic trigger. To enable rapid and cost-effective testing, a lateral flow device was developed and verified for excellent sensitivity and specificity. Urinary CA1 provides a sensitive and practical readout of intravascular hemolysis suitable for point-of-care testing globally.

## Introduction

Red blood cells (RBCs) survey all vascularized tissues and become exposed to various mechanical, chemical, metabolic, infective, and immunological stresses.^1,2^ With limited defenses, RBCs may accumulate damage to the point of rupture (hemolysis), a sentinel of disease-related triggers affecting ~800 million people. Some presentations (eg, thrombotic thrombocytopenic purpura and hemolytic uremic syndrome) are immediately life-threatening and mandate rapid testing.^3^ Hemolysis may appear as early as the neonatal period: physiologically during fetal RBC replacement^4^ or pathologically with birth complications such as sepsis^5–7^ and immune reactions.^8–11^ Lifelong predisposition can arise from genetic traits^12–15^ affecting hemoglobin (Hb),^16^ the cytoskeleton,^17,18^ or metabolism^19–21^; for example, glucose-6-phosphate dehydrogenase deficiency renders RBCs susceptible to rupture upon oxidative stress arising from certain foods^22^ or medications.^23^ In low- and middle-income countries,^24–26^ common hemolytic triggers include bacterial^27^ and protozoan (notably malaria^28^) infection. Inflammation can further exacerbate hemolysis through autoimmune hemolytic anemia^29^ and complement activation,^30^ as observed in paroxysmal nocturnal hemoglobinuria^31^ or in response to viral infection.^32,33^ Given the global burden, cost-effective diagnosis using point-of-care testing is a recognized priority.

Currently, multiple markers are used to infer intravascular hemolysis because individual tests have their limitations. The most direct markers are cytoplasmic proteins released from ruptured RBCs, but these are prone to false-positive readings if samples are drawn, stored, or processed improperly. A rise in serum lactate dehydrogenase (LDH) may not be specific to RBC rupture because it is present in many other cells.^34,35^ Plasma-free Hb (PFH)^36^ is RBC specific, but binding to haptoglobin (Hp)^37^ and salvage by hepatic and splenic macrophages reduces its sensitivity to detect hemolysis. Although Hp depletion can estimate this buffering effect, interpretation requires baseline levels of this liver glycoprotein^38,39^ and is confounded by age, genetics, and hepatic disease.^40^ In newborns, for example, hepatic immaturity depresses Hp production,^41,42^ thereby risking misclassification of low Hp as hemolysis.^43,44^ Another inverse marker is blood Hb, which may fall post-hemolysis,^45,46^ but interpretation hinges on knowing prior levels to distinguish RBC rupture from suppressed production. Indirect markers of hemolysis include raised reticulocyte count, but this delayed response can be nonspecific.^47,48^ Bilirubin measurements give a delayed readout of hemolysis by reporting heme catabolism, but tend to associate with extravascular RBC rupture^49^ and are influenced by variation in clearance.^50^ Although bilirubin levels can be detected transcutaneously, this approach is inaccurate with darker skin tones and leads to racial bias.^51,52^ Moreover, false positives can arise when hepatic clearance of conjugated bilirubin is inadequate, common in early life, or in nonhemolytic conditions such as Crigler-Najjar syndrome.^53^ The direct antiglobulin test (DAT), designed to detect immune sensitization to RBC damage,^54–56^ also has a high false-positivity rate.

An overarching limitation of blood-based hemolysis assays is their poor fit for cost-effective point-of-care testing. A practical alternative is to measure markers excreted in urine. Although LDH and the Hb-Hp complex are too large to cross the glomerular filter, some filtration of unbound Hb may occur after Hp depletion with severe hemolysis, but this is largely reclaimed by tubular mechanisms.^57^ This process can be detected several days later by measuring urinary hemosiderin,^58^ a product of iron metabolism retained in sloughed epithelial cells. We propose that a more sensitive and direct urinary marker of hemolysis is carbonic anhydrase 1 (CA1), previously reported by us in a small cohort of neonates in intensive care.^59^ After Hb isoforms, CA1 is the most abundant protein in the RBC cytoplasm (~4 g/L in the blood of adults and ~1 g/L in the blood of newborns)^60,61^; therefore, rupture can lead to a significant rise in plasma CA1 levels from a negligible baseline.^61,62^ Unlike Hb, plasma CA1 is not appreciably buffered or reclaimed,^63^ which amplifies its sensitivity to detect RBC rupture. Blood-borne CA1 crosses the glomerular filter more readily than Hb^60,64,65^ and, in the absence of tubular reclamation,^57^ emerges in urine. Here, we quantify urinary CA1 excretion across newborns, children, and adults spanning a range of hemolytic severity and geography to validate CA1 as a sensitive marker of intravascular hemolysis and to demonstrate its detection using a lateral flow device (LFD) suitable for routine, low-cost point-of-care testing.

## Methods

### Oxford cohort

Consenting adults self-reporting as healthy provided reference samples (exclusion: active menstruation). Samples obtained from consenting adult participants attending the inherited anemias clinic in Oxford University Hospitals included sickle cell disease (SCD) in steady-state and a number of other disorders less likely to cause intravascular hemolysis (exclusion: thalassemia). Urine collection from healthy donors was approved by Oxford University Interdivisional Research Ethics Committee. Patients with anemia attending the clinic at Oxford University Hospitals were recruited under approval from the National Health Service (NHS) Research Ethics Committee (REC) 13/WA/0371.

### London cohort

Newborns admitted to the intensive care of Evelina’s Children’s Hospital for at least 5 days provided daily urine samples up to postnatal day 10. Eligible newborns were screened near birth, and consent for recruitment was sought from their parent(s) or legal guardian(s). The study was approved by NHS REC 22/EM/0103.

### Bangladesh cohort

Participants were a subset of patients enrolled in an observational clinical trial^66^ and an interventional clinical trial assessing paracetamol as adjunctive therapy in severe falciparum malaria.^67^ Eligible participants with complicated or uncomplicated falciparum malaria, aged ≥ 10 years, were enrolled at Chattogram Medical College Hospital (Chattogram, Bangladesh) and Ramu Upazilla Health Complex (Ramu, Bangladesh). Informed consent was obtained from the patient or a legally acceptable representative. The study was approved by the Oxford University Tropical REC (OxTREC 21-11/07-12), Chittagong Medical College Ethics Committee (CMC/PG/2014/19), and the Bangladesh Medical Research Council (BMRC/NREC/2010-2013/1208).

### Peru cohort

Participants were recruited among adults attending rural health centers in San José de Lupuna, Santa Clara, Santo Tomas, and Varillal of the Loreto region, where malaria is also routinely tested.^68,69^ Participants provided informed consent and completed a health-related questionnaire (exclusion: renal disease, cancer, and practice of high-intensity sports). The study was approved by the Institutional Ethics Committee of Universidad Peruana Cayetano Heredia (CIEH-UPCH 143-06-23, SIDISI 208596).

Supplemental Methods provide details on sample collection and storage, immunoblotting and enzyme-linked immunosorbent assay (ELISA), LFD development, and statistical analyses.

## Results

### Urinary CA1 and Hb excretion across a spectrum of hemolytic severity

**Figure 1:**
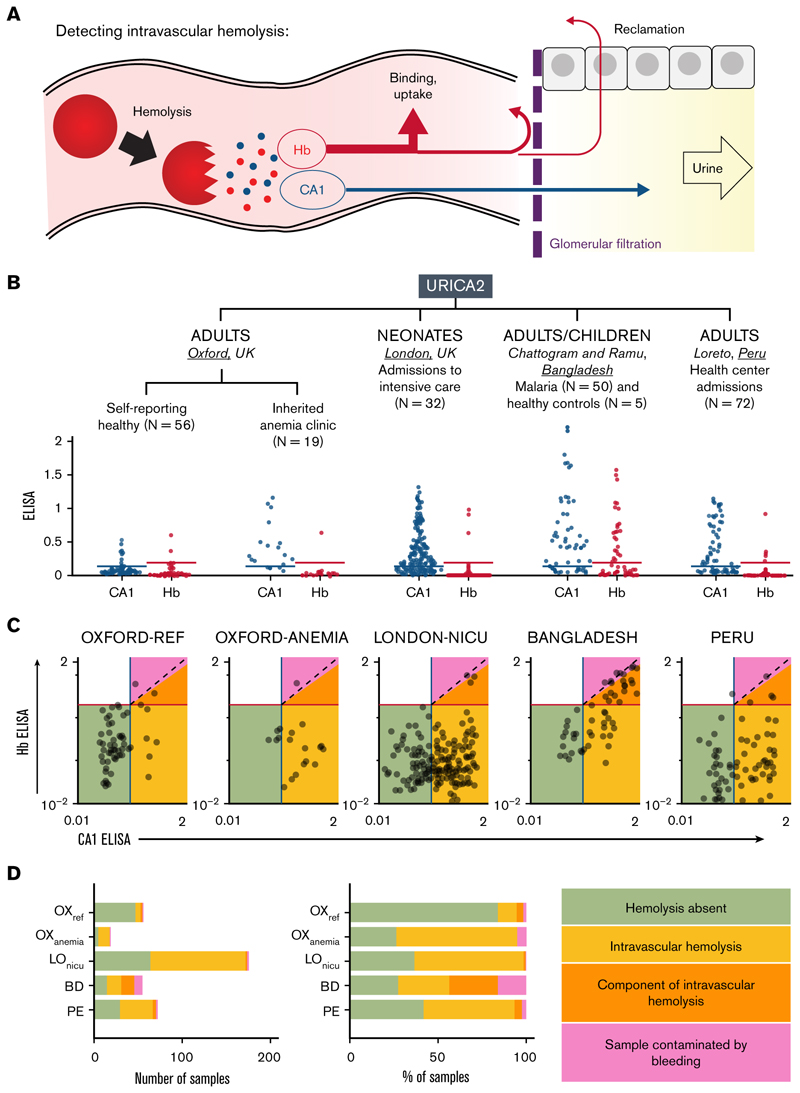
Relating urinary CA1 and Hb excretion. (A) Hypothesis: intravascular hemolysis releases Hb and CA1 stoichiometrically, but extensive binding, uptake, and reclamation of Hb reduce its excretion, whereas CA1 passes more freely into urine. (B) Model tested by relating CA1 and Hb excretion in cohorts spanning a range of hemolytic risk in 4 arms of the URICA2 study. Urine CA1 or Hbα ELISA quantification, normalized to positive control (10 000× diluted blood lysate). Thresholds were defined using the reference cohort after removing outliers (Grubb method). (C) Relationship between ELISA CA1 and ELISA Hb quantification on a log-log plot. Samples were divided into 4 groups using thresholds defined for CA1 and Hb. CA1-positive/Hb-negative samples are highly likely intravascular hemolysis (yellow region). Dually negative samples are likely nonhemolyzing participants (green region). Samples where CA1 signal exceeds the Hb signal by at least 33% were deemed to have at least a component of intravascular hemolysis (orange region). Samples of comparable Hb and CA1 signals were deemed to be contaminated with blood (red region). (D) Breakdown of samples by number or percentage. BD, Bangladeshi samples; LO_NICU_, samples from the London NICU; OX_ANEMIA_, samples from patients attending inherited anemia clinic; OX_REF_, Oxford reference samples; and PE, Peruvian samples.

Intravascular RBC rupture releases Hb and CA1, of which, the former is reclaimed, and the latter is filtered across the glomerular barrier (Figure 1A). We postulate that this event can be detected as CA1-positive, Hb-negative urine. A confounding source of CA1 is urogenital bleeding (eg, menstruation and injury), but this would be distinguishable by a stoichiometric release of Hb. To relate urinary CA1 and Hb excretion with hemolytic severity across diverse populations and triggers, URICA2 (Urinary excretion of Carbonic Anhydrase Study 2; supplemental Figure 1A) enrolled 234 participants across the United Kingdom (Oxford and London), Bangladesh (Chattogram and Ramu), and Peru (Loreto region). In Oxford, 75 adults were recruited, including 56 self-reported healthy volunteers (supplemental Table 1) and 19 patients predisposed to hemolysis attending inherited anemia clinics (supplemental Table 2). To examine early postnatal dynamics, 32 newborns were recruited among neonatal intensive care unit (NICU) admissions (supplemental Figure 1B; supplemental Tables 3 and 4). A wide range of hemolytic risk was covered by the Bangladeshi cohort of children (age of ≥ 10 years) and adults, comprising 30 complicated and 20 uncomplicated cases of *Plasmodium falciparum* malaria, alongside 5 healthy controls (supplemental Figure 1C; supplemental Table 5). In addition, this cohort is well suited to assess blood contamination as a confounder because urine samples from 28 complicated and 3 uncomplicated cases were collected via a Foley catheter, a method that can cause urogenital bleeding (supplemental Figure 1D). Finally, to evaluate urinary CA1 excretion in an undifferentiated group of conditions, urine samples were obtained from 72 adults attending health clinics for various reasons in Peru’s Loreto region, where *P vivax* malaria is endemic (supplemental Figure 1E; supplemental Table 6).

Urine samples were assayed for CA1 and Hbα by ELISA (Figure 1B). Signals were normalized to calibration samples spiked with freshly prepared blood lysates (1:10 000 volume-to-volume dilution), which release CA1 and Hbα stoichiometrically. Hence, comparable CA1 and Hbα signals indicate blood contamination, whereas a dominant CA1 signal argues for intravascular hemolysis. Based on the spread of CA1 and Hbα in spiked urine samples (supplemental Figure 2), a CA1 signal at least 50% stronger than paired Hbα was defined as arising at least partly from intravascular hemolysis. Positivity thresholds for CA1 and Hbα were set at the upper bound of reference urine samples after outlier removal by Grubbs test (Figure 1B). Applying these criteria, the samples were classified as nonhemolyzing (CA1^−^/Hb^−^; green in Figure 1C), intravascular hemolysis (CA1^+^/Hbα^−^; yellow in Figure 1C), with a component of intravascular hemolysis (CA1^+^ with Hbα signal <67% of CA1; orange in Figure 1C), or blood contaminated (CA1^+^ with Hbα signal >67% of CA1), which is ambiguous because it can mask a true hemolytic event. No sample was Hb^+^/CA1^−^, which verifies that the release of Hb from ruptured RBCs must be accompanied by CA1. The reference group excreted mostly CA1^−^/Hb^−^ urine samples (84%). Patients attending the anemia clinic produced Hb^−^ urine samples spanning a range of CA1 signal. Among 175 samples obtained from 32 newborns, 108 (62%) were CA1^+^, only 3 were also Hb^+^ and just 1 classified as strongly contaminated by blood. In the Bangladeshi cohort, all healthy controls were CA1^−^/Hb^−^, 31 malaria cases (55%) had at least a component of intravascular hemolysis, and 9 urine samples (16%) were contaminated by bleeding. In the Peruvian cohort, 40 samples (56%) were classified as intravascular hemolysis, 2 (3%) as contaminated, and the remainder as CA1^−^/Hb^−^. Figure 1D summarizes the classification by urinary CA1/Hb status.

**Figure 2:**
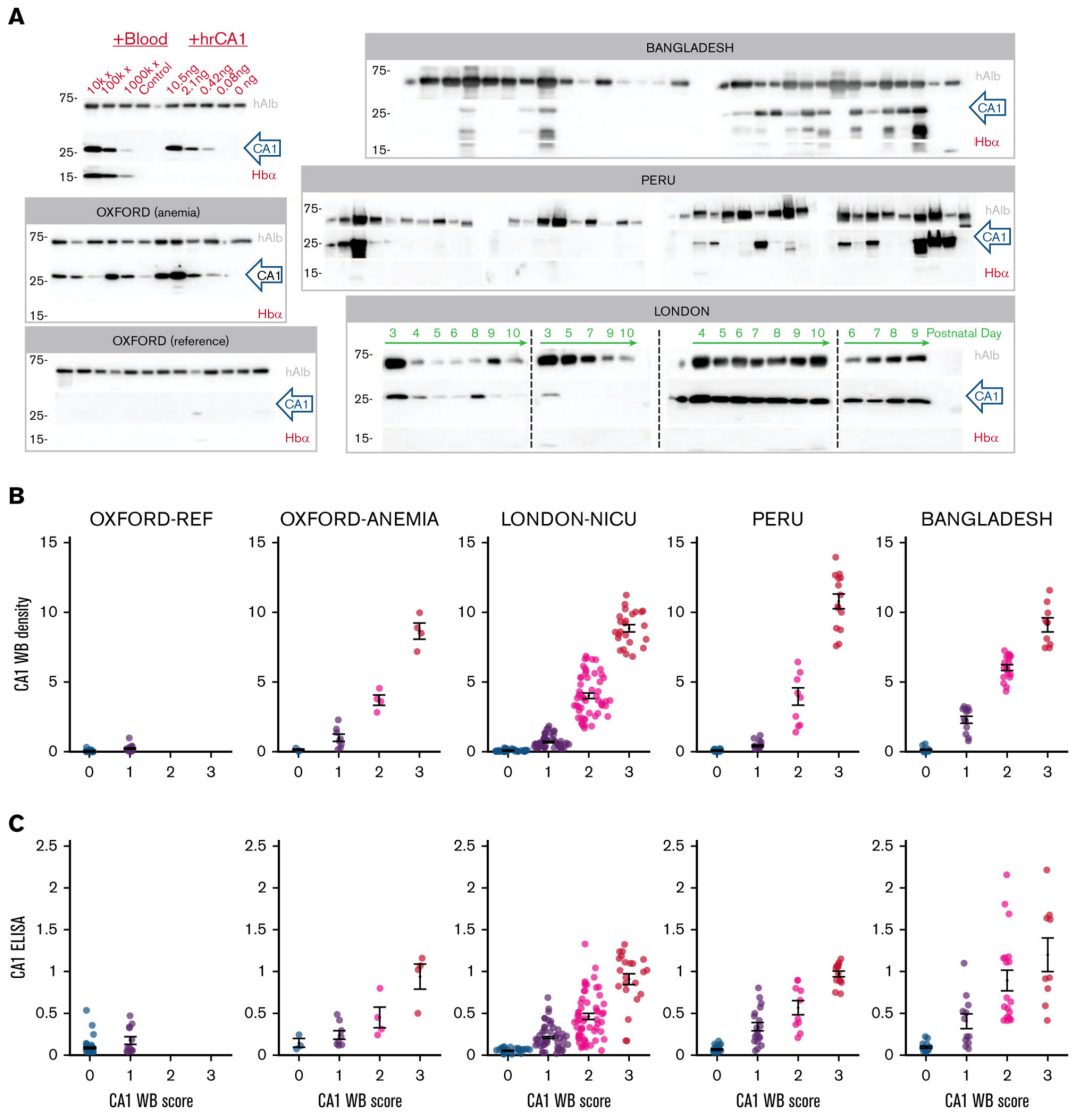
Verifying CA1 and Hb immunoreactivity on western blot. (A) Western blots (WBs) for hAlb, CA1, and Hbα in the Oxford, Bangladeshi, Peruvian, and London cohorts. Further shown is a concentration response for RBC lysates and human recombinant CA1 (hrCA1) diluted in CA1-free adult urine. Adult samples were obtained on 1 occasion; neonatal samples were obtained longitudinally on the days indicated, over the first 10 postnatal days of life. (B) Relationship between CA1 densitometry and its visual score (0-3) across the 5 study groups. Color coding relates to the visual score for each WB, from not detectable (0, blue) to highest intensity (3, red). (C) CA1 ELISA quantification, normalized to positive control (10 000× diluted blood lysate), vs WB visual score. Summary statistics show mean ± standard error of the mean (SEM). hAlb, human albumin; rhCA1, recombinant human CA1.

Each urine was also analyzed by western blot (Figure 2A; supplemental Figures 3-14). CA1 and Hb bands were scored visually: 0 (unambiguously negative), 1 (faint signal), 2 (strong signal) and 3 (very strong signal), relative to RBC-spiked controls (Figure 2B). Western blot scores broadly agreed with the ELISA results (Figure 2C). Reference urine samples were mostly CA1^−^/Hb^−^, with occasional low-level CA1 (score 1). In contrast, patients from the anemia clinic, neonates in intensive care, participants with malaria infection in Bangladesh, and health clinic attendees in Peru excreted a range of CA1 in urine, mostly without proportional Hb. Exemplar time courses from 4 newborns illustrate distinct patterns, namely unstable CA1 excretion, an early low-amplitude transient, and sustained high-level CA1 excretion.

In summary, urinary CA1 levels ranged from negligible in healthy volunteers, to elevated across hemolytic predispositions, with most CA1-positive urine samples showing markedly lower or absent Hb, consistent with intravascular RBC rupture followed by glomerular separation of CA1 from Hb.

### Urinary CA1 excretion in inherited anemia correlates with blood markers of hemolysis

**Figure 3:**
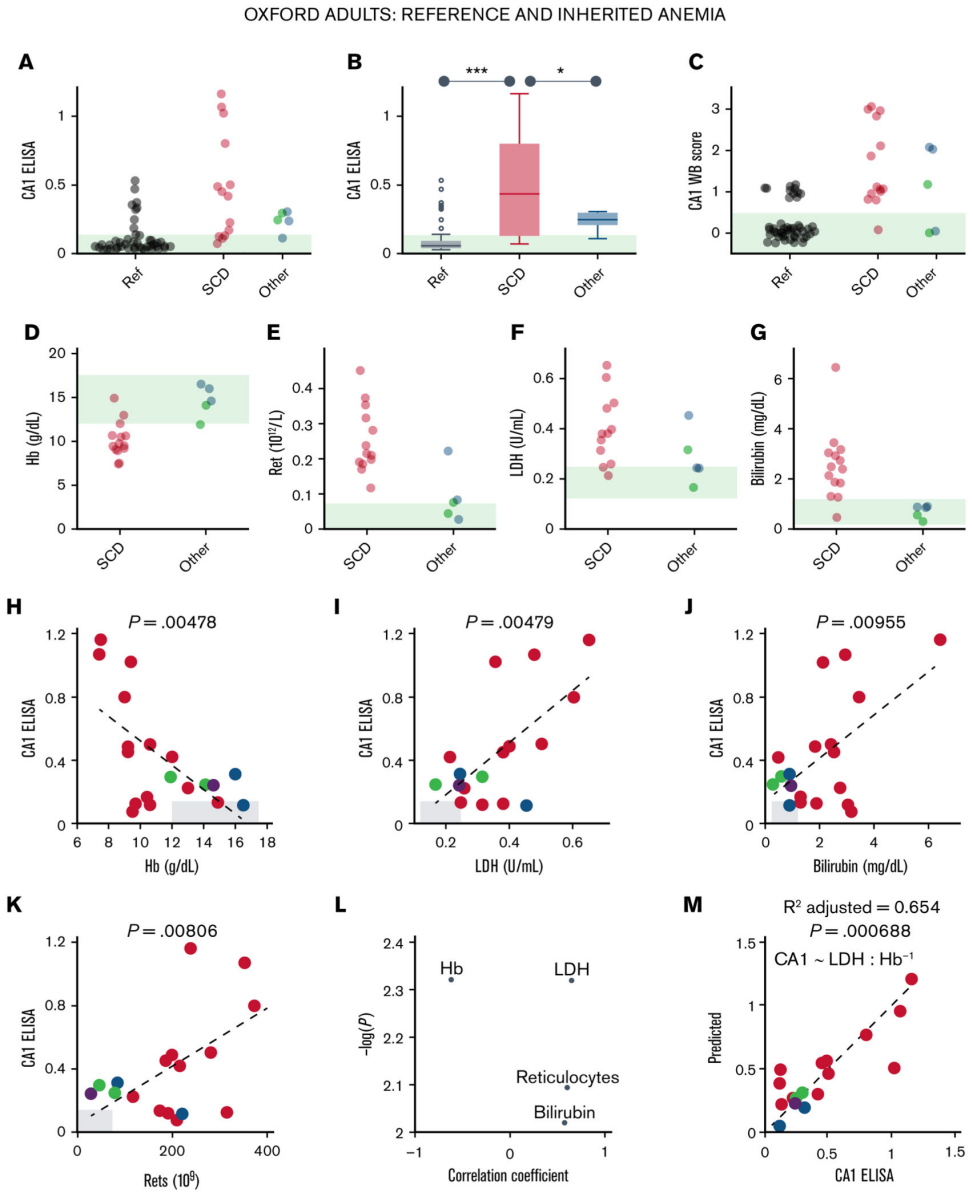
Urinary CA1 excretion is raised in inherited anemias linked to intravascular hemolysis. (A) Urinary CA1 excretion measured by ELISA for self-reported healthy (“Ref”) participants, patients with SCD, and patients with nonhemolytic anemia (hemochromatosis, Diamond-Blackfan anemia, hereditary spherocytosis, and Southeast Asian ovalocytosis). (B) Significant increase in CA1 excretion in SCD (1-way analysis of variance). *P < 0.05, ***P < 0.001. WB score for CA1 (C), blood Hb (D), reticulocyte count (E), LDH (F), and bilirubin (G). Correlation (Pearson test) between CA1 ELISA and blood Hb (H), LDH (I), bilirubin blood markers (J), and reticulocytes (K). (L) Relationship between Pearson correlation coefficient and significance. (M) Linear mixed-effects model for Hb and LDH accurately predicts CA1 signal. Ref, reference; Ret, reticulocyte.

Next, the diagnostic utility of urinary CA1 excretion was assessed by correlation against a panel of blood markers of hemolysis. Compared to self-reporting healthy volunteers, urine samples from participants recruited at the inherited anemia clinic spanned a range of urinary CA1 signal on ELISA (Figure 3A-B) and western blot (Figure 3C). Among admissions, 14 had SCD in steady state, for whom intravascular hemolysis is expected, and 5 had conditions less likely to cause intravascular hemolysis: Diamond-Blackfan anemia, hemochromatosis, hereditary spherocytosis (2 cases), and Southeast Asian ovalocytosis. All samples were included in the analysis. Participants with SCD excreted significantly more CA1 than healthy controls and the other anemia cases (Figure 3B), and had reduced blood Hb and raised reticulocyte count, serum LDH, and bilirubin, consistent with intravascular hemolysis (Figure 3D-G). Urinary CA1 correlated significantly with these blood markers (Figure 3H-L), with strongest associations observed for blood Hb and LDH. A linear mixed-effects model provided an excellent fit using these variables (Figure 3M). Urinary CA1 excretion is, therefore, significantly associated with blood markers of hemolysis in an at-risk adult group with inherited anemia.

### Urinary CA1 excretion stratifies newborns by hemolytic severity

**Figure 4:**
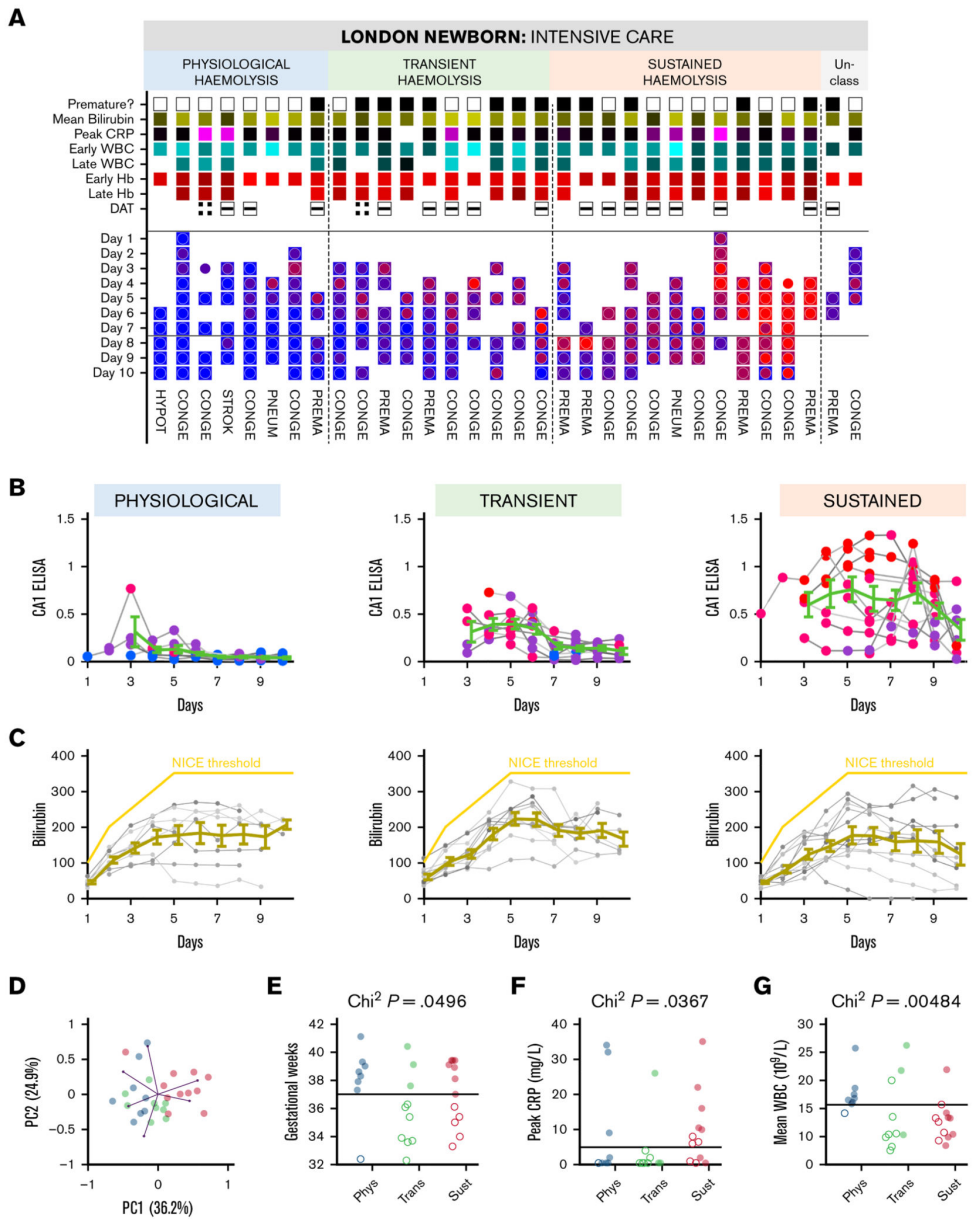
Urinary CA1 excretion classifies newborns and is associated with infection markers. (A) Urinary CA1 excretion in the first 10 days of life in 32 neonates, grouped into 3 patterns (2 participants were unclassified due to insufficient sampling). The color of the square symbols relates to the ELISA CA1 score, from 0 (blue) to the highest (red). The color of circle symbols relates to WB densitometry, from 0 (blue) to the highest (red). Ranking within group is by mean ELISA CA1 level. Further shown is prematurity (black denotes <37 weeks), mean bilirubin level normalized to the NICE threshold for phototherapy (yellow denoting higher level), peak CRP during the 10-day period, early WBC and Hb are the first records, late WBC and Hb are the mean of subsequent measurements, and DAT results. The bottom portion shows the clinical reason for admission. (B) Time courses of the CA1 ELISA results in the 3 hemolysis groups. Color coding relates to WB score, from 0 (not detectable, blue) to 3 (highest, red). (C) Time courses of bilirubin. The yellow line shows the threshold for indicating phototherapy according to the NICE guidelines. Mean ± SEM for available data. (D) Unsupervised principal component analysis of standardized data. Relationship between neonate CA1 classification and gestational age (weeks) (E), peak CRP in the first 10 days of life (F), mean WBC (G) in the first 10 days of life. *P* values refer to the χ^2^ test. CONGE, congenital condition; DAT, direct antiglobulin test; HYPOT, hypotonia NICE, National Institute for Health and Care Excellence; PC, principal component; Phys, physiological; PNEUM, pneumothorax; PREMA, premature birth; STROK, stroke; Sust, sustained; Trans, transient.

The ability of urinary CA1 excretion to differentiate newborns by hemolytic severity was evaluated over early postnatal life, during which hemolysis can range from physiological to pathological. To enable longitudinal sampling over a period of major developmental changes, sampling was conducted at NICU. Thirty-two neonates were enrolled, including 20 with a congenital anomaly, 2 with pneumothorax, 1 with stroke, and 1 with hypotonia. Fourteen babies were born prematurely (<37 weeks). Up to daily urine samples were collected over the first 10 postnatal days (median of 6 samples per participant), alongside blood tests and skin bilirubinometry. Figure 4A illustrates the prematurity status, mean bilirubin level (normalized to National Institute for Health and Care Excellence [NICE] threshold), peak level of C-reactive protein (CRP) during the first 10 days, DAT result, and the initial and time-averaged white blood cell count (WBC) and blood Hb. Given that all but 3 urine samples were Hb-negative, CA1-positive urine samples likely reflect intravascular hemolysis (Figure 1C). To maximize the use of data without imputation, CA1 status was compared in 2 periods (days 1-7 vs days 8-10) for which all but 2 infants contributed data. Unsupervised clustering of CA1 time courses identified 3 patterns: (1) “physiological hemolysis”, with no more than low signal in the first week and CA1-negativity thereafter; (2) “transient hemolysis”, with significant signal in the first week and reduced but detectable signal thereafter; and (3) “sustained hemolysis”, with strong or rising signal. CA1 levels in the last group approached those observed in hemolyzing adults with SCD. Despite these distinct CA1 trajectories (Figure 4B), bilirubin time courses were not significantly different between the groups (Figure 4C) and provided only limited information to differentiate infants. Principal component analysis (PCA) of 9 variables indicated that early and late CA1 contributed most to the primary axis, whereas bilirubin contributed the least (supplemental Figure 15B-C), consistent with bilirubin’s limited specificity and delayed kinetics as a hemolysis marker. After pruning highly correlated variables, a PCA of early and late CA1, mean bilirubin, mean CRP, mean blood Hb, and mean WBC revealed a clear separation of newborns by CA1 group (Figure 4D).

Further analyses revealed an association between CA1 grouping and gestational age. All but 1 infant in the physiological group were full-term, whereas preterm birth was significantly associated with higher CA1 excretion (Figure 4E). Peak CRP of ≥5 mg/L was also enriched in groups with higher CA1 excretion (Figure 4F). Decision tree analysis identified mean WBC as the strongest splitter of CA1 groups (supplemental Figure 15D), recapitulated in Figure 4G: physiologically hemolyzing full-term infants had WBC in the range of 15 × 10^9^/L to 25 × 10^9^/L, whereas the transient and sustained groups showed lower counts. Although a WBC of 15 × 10^9^/L is not considered leukopenic, the inverse association between CA1 excretion and WBC in combination with elevated CRP level may indicate increased risk of neonatal sepsis.

### Urinary CA1 excretion correlates with PFH in malaria infections

**Figure 5:**
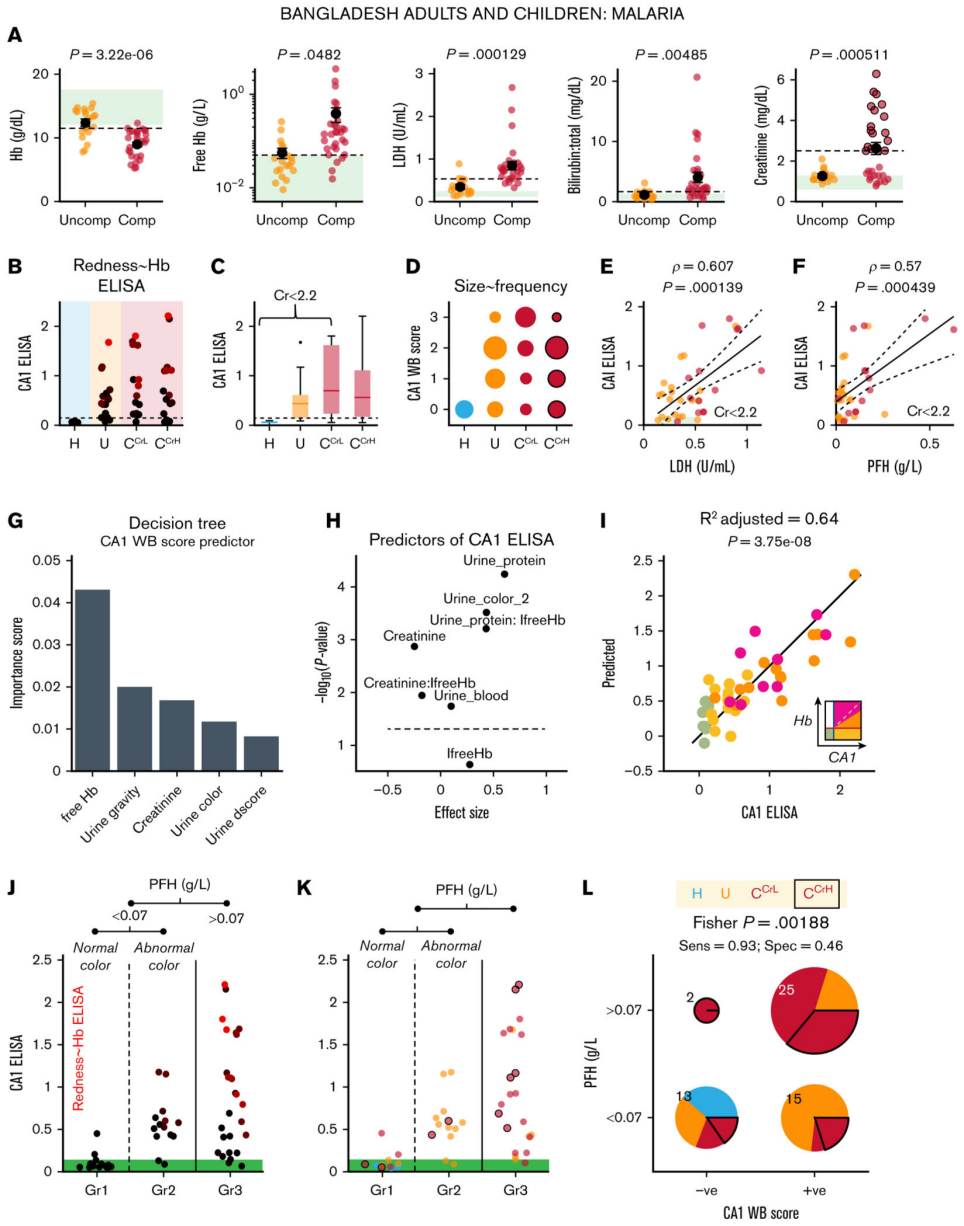
Urinary CA1 excretion is a sensitive marker of intravascular hemolysis. (A) Blood Hb, PFH, LDH, total bilirubin, and creatinine levels in uncomplicated and complicated cases of malaria in the Bangladeshi cohort. The green box indicates the reference range. The dashed line indicates the threshold that best separates the 2 populations (maximal Youden index after receiver operating characteristic analysis). (B) Relationship between urinary CA1 ELISA and patient group. The ELISA data are color-coded in proportion to the Hb ELISA signal. (C) Averaged data showing trends. (D) Relationship between the WB score of urinary CA1 and patient group. Relationship between CA1 ELISA and LDH (E) or PFH (F) for patients with creatinine level <2.2 mg/dL to exclude renal failure. The lines indicate 95% confidence interval of best fit. (G) Decision tree analysis against the WB score of urinary CA1 identified PFH as the most important predictor. (H) Effect size and significance of the predictors, highlighting PFH (interactions with urine protein score and normal/abnormal color) as the most important predictor of urinary CA1 excretion. (I) Regression analysis produced a highly significant model for estimating CA1 ELISA signal. Color coding denotes the class of urine, as determined from the Hb/CA1 relationship. (J) Urinary CA1 ELISA signal broken down by PFH thresholded at 0.07 g/L and further by normal or abnormal urine color for PFH level <0.07 g/L. The symbol color denotes the Hb ELISA signal (red, higher). (K) Data are replotted, with the color denoting the patient groups in panel A. (L) PFH (threshold, 0.07 g/L) and the WB score of CA1 (negative/positive) for all samples show a significant association. C^CrH^, complicated malaria with high creatinine level; C^CrL^, complicated malaria with low creatinine level; Comp, complicated malaria case; Cr, creatinine; Sens, sensitivity; Spec, specificity; U and Uncomp, uncomplicated malaria cases.

The performance of urinary CA1 as a hemolysis marker was tested in *P falciparum* malaria infection, a setting of severe RBC rupture (supplemental Figure 16A) with potential confounders, including renal failure and catheter-related urogenital bleeding among unconscious patients (supplemental Figure 16B). Compared to uncomplicated cases, complicated malaria was associated with reduced blood Hb and elevated PFH (mostly >0.05 g/L), LDH (>250 U/L), bilirubin, and creatinine (>3 mg/dL; Figure 5A). To account for the effect of renal function on urinary readouts, complicated cases (C) were stratified by creatinine at 2.2 mg/dL into low (C^CrL^) and high (C^CrH^) subgroups.

Urinary CA1 levels measured by ELISA, were lowest in healthy controls, intermediate in uncomplicated malaria, and highest in C^CrL^. CA1 excretion in the C^CrH^ group displayed the greatest variability, consistent with renal function affecting CA1 handling (Figure 5B). Western blot scoring followed the same pattern (Figure 5C). Strong CA1 signals sometimes coincided with urinary Hb, raising the possibility of blood contamination (supplemental Figure 16C). Blood markers PFH and LDH correlated closely and separated uncomplicated from complicated malaria at ~0.05 g/L of PFH and ~535 U/L of LDH, but did not distinguish C^CrL^ from C^CrH^ (supplemental Figure 16C). To test for mechanistic link between urinary CA1 and blood markers of hemolysis without confounding factors due to renal function, correlations were assessed in participants with low creatinine; this verified that urinary CA1 correlated strongly with both LDH and PFH (Figure 5E-F).

Decision tree analysis identified PFH (thresholded at 0.07 g/L) as the strongest predictor of the western blot score of urinary CA1 (Figure 5G). Additional predictors were included in a regression model (Figure 5H), and log transformation was applied to PFH to account for its spread. This model produced an excellent fit to all participants, without overfitting or collinearity (variance inflation factor <1.5), using PFH, urine color (normal/abnormal), urine protein (score 0-3), and interactions thereof (Figure 5I). In accordance with decision tree analysis, classifying urine samples by PFH (at 0.07 g/L) and then, among low-PFH samples, by urine color (normal/abnormal), yielded 3 groups (Figure 5J-K): Group-3 (Gr3; PFH ≥0.07 g/L), in which most participants excreted urinary CA1 and were classified as hemolyzing, Group-1 (Gr1; PFH <0.07 g/L with normal urine color), with mostly subthreshold CA1 (nonhemolyzing), and Group-2 (Gr2; PFH <0.07 g/L with abnormal urine color), with generally elevated CA1 excretion despite low PFH. A contingency table analysis of urinary CA1 (western blot score ≥ 1 vs 0) against PFH showed a strong association with high sensitivity (93%) but modest specificity (46%; Figure 5L). In summary, urinary CA1 is mechanistically linked to elevated PFH level in malaria. Most cases of raised PFH also excreted CA1 (ie, bona fide hemolysis) but in some instances, urinary CA1 excretion occurred without raised PFH. Thus, urinary CA1 excretion can detect cases of hemolysis that PFH is unable to resolve, likely because of the strong buffering effect on plasma Hb that attenuates its sensitivity.

### Urinary CA1 excretion correlates with inflammatory markers

**Figure 6:**
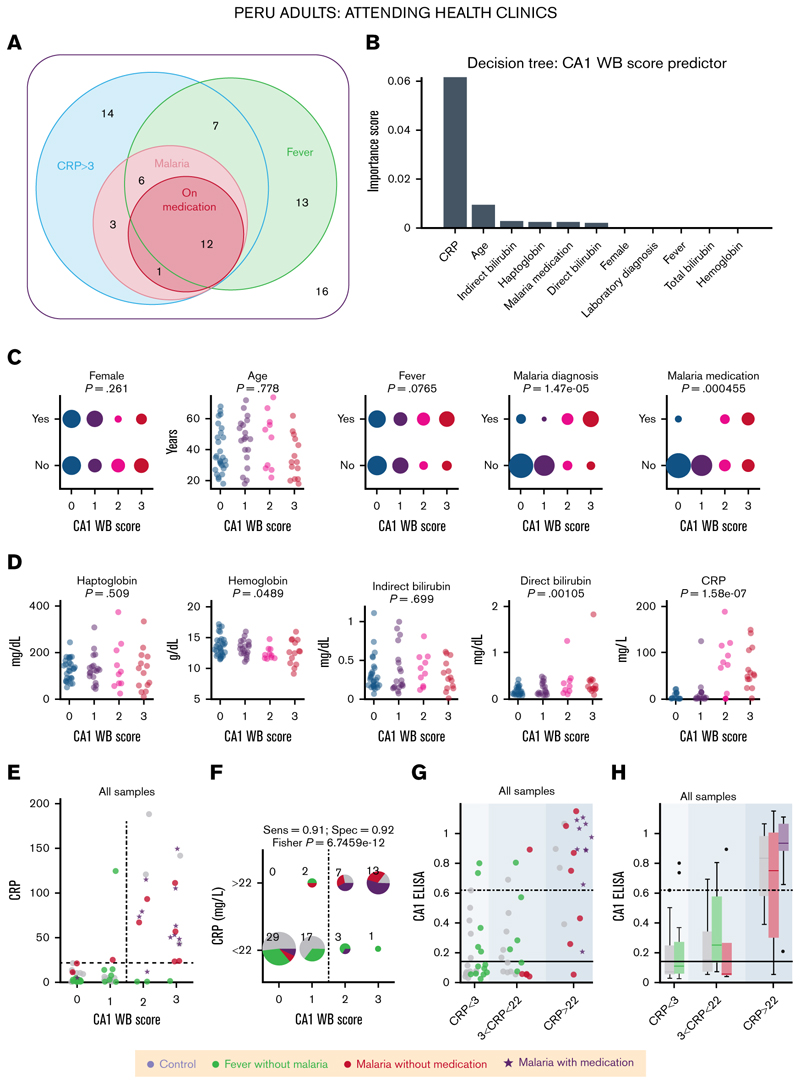
Urinary CA1 excretion is a marker of inflammatory state. (A) Euler diagram showing the classification of Peruvian participants by CRP status, malaria diagnosis, malaria medication, and fever. (B) Result of the decision tree analysis showing the greatest influence of CRP on the WB score of urinary CA1. (C) Relationship between demographic or clinical information and urinary CA1 score. Significance testing by Spearman correlation. (D) Relationship between blood measurements and urinary CA1 score. Significance testing by Spearman correlation. (E) Relationship between CRP and the WB score of urinary CA1. The symbols encode participant status (control, fever without malaria, malaria without medication, and malaria with medication). (F) Thresholding CRP at 22 mg/L confirms >90% specificity and sensitivity. (G) CA1 ELISA result broken down by CRP level and disease status, and reploted as box plots (H). Sens, sensitivity; Spec, specificity.

The results thus far demonstrated the utility of urinary CA1 as a marker in specific cohorts, including inherited anemia, admissions to NICU, and malaria. To study a more undifferentiated patient group, analyses were performed on samples from 72 adults attending 4 rural health clinics in the Peruvian Loreto region for various medical reasons (Figure 6A). Routine microscopy identified malarial infection in 7 females and 15 males, likely *P vivax* that infects immature RBCs and causes moderate hemolysis (supplemental Figure 17A). The cohort included nonmalarial febrile cases and individuals without evidence of infection, thereby spanning a range of inflammation, a hemolytic trigger. Correlations were analyzed between urinary CA1 and blood Hb, direct and indirect bilirubin, Hp, and CRP, information on fever, malaria test result, and antimalarial prescriptions (chloroquine/primaquine). Malaria diagnosis associated with lower Hp and higher direct bilirubin levels, consistent with hemolysis, although effect sizes were small, with most values in the reference range (supplemental Figure 17B-E). In contrast, CRP was markedly elevated among malaria-positive cases, with all exceeding 3 mg/L; a subset of nonmalarial cases also had high CRP (supplemental Figure 17F).

Decision tree analysis using the western blot score of urinary CA1 as the outcome identified CRP level as the strongest predictor (Figure 6B). CA1 score showed no association with age or sex but correlated positively with malaria diagnosis and treatment status: 18 of 22 malaria cases displayed CA1 immunoreactivity on western blot (Figure 6C). Consistent with the weak linkage between Hp and malaria, CA1 did not correlate with Hp. Instead, CA1 correlated positively with direct bilirubin and with CRP (Figure 6D), aligning with the decision tree result. Applying a CRP threshold of 22 mg/L separated participants into CA1^low^ (western blot score, 0-1) and CA1^high^ (score, 2-3) groups with >90% sensitivity and specificity (Figure 6E-F). CRP^high^/CA1^high^ individuals were typically malaria-positive, whereas CRP^low^/CA1^low^ were either nominal controls or nonmalarial febrile cases. Analyses using CA1 ELISA yielded similar conclusions (Figure 6G-H), with CA1 positivity linked to higher inflammatory states, particularly with drug-treated malaria.

Overall, substantial urinary CA1 excretion tracked systemic inflammation and outperformed Hp and bilirubin for the purpose of stratifying patients by disease severity and hemolytic risk.

### Urinary CA1 can be accurately detected on a LFD

**Figure 7:**
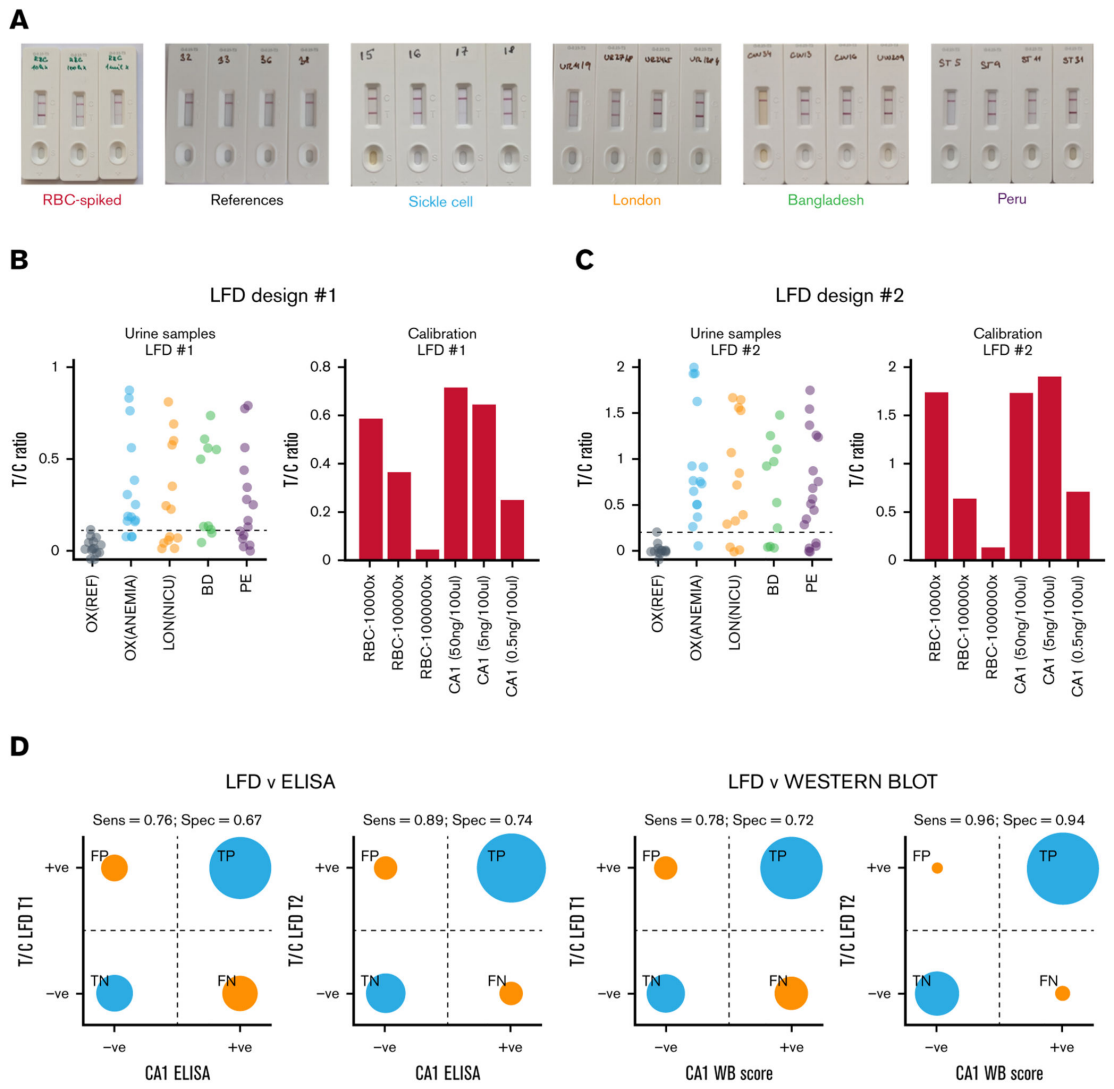
LFD for urinary CA1. (A) Exemplar LFD test results from the 5 participant cohorts plus calibration standards and densitometric analysis of their T and C bands. (B) T:C ratios and calibrations for the first and (C) second LFD design. (D) Assessment of sensitivity and specificity of LFD reading against laboratory ELISA CA1 test and (E) against laboratory western blot analysis. C, control band intensity; FN, false negative; FP, false positive; T, test band intensity; TF, true negative; TP, true positive.

A urine-borne biomarker is suitable for routine point-of-care testing using LFDs (Figure 7A). Two designs were manufactured, differing in the concentration of capture antibodies (fivefold higher in design 2). These were tested using representative samples from all study arms (Figure 7B-C). LFD performance was benchmarked against ELISA (supplemental Figure 18A) and western blot (supplemental Figure 18B). The antibodies used for the LFD and ELISA did not cross-react with CA2, an isoform with the closest (60%) homology to CA1, nor with a mixture of CA isoforms expressed in HT29 cancer cells (supplemental Figure 19). The threshold for a positive test band:control band (T:C) intensity ratio was taken as the upper limit of the reference samples that had a western blot score of 0 or subthreshold ELISA signal. Sensitivity and specificity reached >90% for design 2 against western blot score, providing a path for point-of-care testing (Figure 7D-E).

## Discussion

Our analysis of 234 participants across diverse geographies shows that urinary CA1 is negligible in controls (reference group, healthy Bangladeshi participants, and malaria-negative Peruvian participants with no fever and low CRP level) and increases with hemolytic risk, with the highest levels in malaria infection and SCD. Results from ELISA, western blot, and a custom LFD were broadly concordant, indicating stable CA1 epitopes across antibody-based assays. CA1 is among the most abundant RBC cytosolic proteins and is minimally buffered or reclaimed upon release into plasma.^63^ Following intravascular RBC rupture, CA1 crosses the glomerular filter and, in the absence of tubular salvage mechanisms, appears in the urine. In contrast, RBC-released Hb can bind to Hp, undergo scavenging by macrophages^70–73^, or reclamation by tubular mechanisms,^74^ therefore its urinary excretion is expected to be negligible in comparison to CA1. A confounding source of urinary CA1 is bleeding (eg, urogenital injury or menstruation) followed by RBC rupture in situ; however, this event is distinguishable by a stoichiometric CA1:Hb rise and was rare in the studied cohorts. Hb-negative urine occurred in >95% of neonatal CA1-positive urine samples, >90% of Peruvian CA1-positive urine samples, and all SCD urine samples supporting an intravascular origin of RBC rupture. The Bangladeshi cohort included a number of Hb-positive urine samples, which may reflect urogenital bleeding in catheterized participants; however, most still showed excess CA1, indicating at least a component of intravascular hemolysis. Only a fifth of the samples were ambiguous (ie, near-stoichiometric CA1:Hb excretion), largely among hospitalized patients. However, these cases would have access to alternative diagnostic methods for hemolysis, and are not the primary beneficiaries of point-of-care testing. Overall, our study provides evidence that the separation of CA1 from Hb in urine is an indicator of intravascular hemolysis proximal to the glomerular barrier.

With minimal systemic processing and a direct urinary readout, CA1 is less affected by maturity and therefore provides a better indication of hemolysis in early postnatal life. Among multiple markers used in the NICU, urinary CA1 contributed most to variation and could stratify infants by hemolytic severity. Notably, information captured by CA1 was not resolved on bilirubin time courses. CA1 testing may help differentiate hyperbilirubinemia caused by hemolysis from that arising from impaired bilirubin clearance (eg, breastfeeding jaundice or Crigler-Najjar syndrome). Its implementation may improve diagnostic accuracy and management, particularly in individuals with darker skin tones, where transcutaneous bilirubin measurements are less reliable. Significantly, sustained CA1 excretion was found to be associated with elevated CRP and reduced WBC values, which may indicate early sepsis risk.^75–77^ The CA1-CRP association in neonates was notably weaker than in adults, but this may reflect developmental influences; for example, not all postnatal inflammatory states result in hemolysis and CRP may spike shortly after birth in the absence of infection.^78,79^ We propose that urinary CA1 screening may aid sepsis surveillance, particularly for gram-negative infections^80^, and flag clinically significant events in low-income settings. Testing in postnatal weeks 1 and 2 could guide better care by classifying newborns by hemolytic state.

Performance of urinary CA1 as a marker of intravascular hemolysis was supported by its relationship with blood markers. In inherited anemias, CA1 correlated positively with LDH and inversely with blood Hb. In falciparum malaria, urinary CA1 tracked PFH: >90% of participants with PFH >0.07 g/L excreted CA1, including complicated cases with catheterization and visible urinary blood, indicating that urinary CA1 is an accurate surrogate of intravascular hemolysis. The absence of urinary CA1 excretion generally coincided with subthreshold PFH (<0.07 g/L), ie, true negative. Critically, a third of participants with malaria infection and sub-threshold PFH excreted urinary CA1 in excess of Hb, consistent with intravascular hemolysis that evades detection by PFH. These urine samples were mostly not bloody, dismissing erythrocyturia as the source of CA1. This apparently higher sensitivity of urine CA1 over PFH to detect hemolysis may reflect scavenging of the latter^70–73^; thus CA1, which is not bound to plasma proteins,^63^ is excreted in proportion to hemolytic severity.

The Peruvian cohort enabled evaluation across inflammatory states, including vivax malaria and nonmalarial fevers. Urinary CA1 separated participants by malaria diagnosis better than total Hb, Hp, or bilirubin and served as a sensitive and specific (>90%) indicator of elevated CRP (>22 mg/L), independent of underlying cause, consistent with inflammation being prohemolytic.^81^ Of the 22 malaria-positive cases, 13 received chloroquine/primaquine, which may trigger hemolysis in at-risk individuals.^82^ Significant hemolysis was observed in all participants with malaria infection receiving medication, but only in ~60% of unmedicated malaria-positive diagnoses. Thus, screening at-risk groups for CA1 could monitor drug-related hemolysis, inform dose titration, and guide alternative therapies,^83,84^ especially in mass drug administration. More broadly, proactive CA1 testing could flag posttransfusion or posttransplant complications or screen potentially fatal events (eg, thrombotic thrombocytopenic purpura/hemolytic uremic syndrome) or exposure-related crises (eg, favism).

In summary, we present evidence for urinary CA1 excretion as a practical marker of intravascular hemolysis and introduce an LFD for point-of-care testing. Advantages include low baseline, intuitive interpretation, and fewer developmental confounders.

## Key Points

- Urinary CA1 excretion detects intravascular hemolysis across diverse conditions.
- A lateral flow device testing urine for CA1 enables rapid detection of hemolysis in resource-limited settings.
