# Supplementary material for: Urinary carbonic anhydrase 1 excretion is a marker of hemolysis-triggering conditions suitable for point-of-care testing
Source: Blood Glob Hematol. 2026 Mar;2(1):None. doi: 10.1016/j.bglo.2025.100058 (PMC7618779; doi:10.1016/j.bglo.2025.100058)

SUPPLEMENTARY FIGURES AND TABLES

Fig. S1: Four arms of the URICA2 study, showing breakdown by age (years) or gestational age (weeks) among male and female participants (no significant differences).

Fig. S2: Determining Hb/CA1 stoichiometry from RBC lysates at different dilutions. The range indicates that a 33% excess of CA1 over Hb would likely to indicate an additional source of CA1, notably a separation of CA1 from Hb arising from glomerular filtration.

Fig. S3 to Fig S14: Western blot analysis for all participant urine samples.

Fig. S15: (A.) Recruitment and procedures on neonates. Yellow squares: urine samples; gray squares: serum and/or transcutaneous bilirubin; red squares: blood test (Hb, full blood count, CRP, DAT). (B.) PCA for all nine metrics recorded. (C.) Contribution of variables towards variation in the cohort. (D.) Decision tree analysis for predictors of the neonatal CA1 group.

Fig. S16: (A.) Breakdown of participants in Bangladesh: healthy controls, uncomplicated malaria (Uncomp), and complicated malaria (Comp). (B.) Breakdown of participants by urinalysis blood score (0-4) along y-axis and hemolysis score (0-12) color-coded from black (negative) to red for patients with and without Foley catheter. (C.) Breakdown of CA1 ELISA by urine blood score. (D) Correlation between LDH and PFH.

Fig. S17: (A.) Breakdown of participants in Peru by health center: Santo Tomas [ST], San José de Lupuna [SL], Santa Clara [SC] and Varillal [V]. Red bars indicate participants who tested positive for malaria infection. (B.) Blood Hb, (C.) haptoglobin, (D.) direct bilirubin, (E.) indirect bilirubin, (F.) CRP in malaria positive and negative diagnoses. Green box indicates reference range. Dashed line indicates threshold that best separates the two populations (maximal Youden index after ROC analysis). Summary statistics show mean±SEM. Significance tested by t-test.

Fig. S18: (A.) Correlation between T/C band ratio and CA1 ELISA signal or (B.) western blot score for the five cohorts.

Fig. S19: (A.) Testing cross-reactivity of the LFD to recombinant CA2 and lysates of HT29 colorectal cancer cells prepared after standard cell culture. (B.) Testing cross-reactivity of the antibodies used for ELISA to recombinant CA2 and lysates of HT29 colorectal cancer cells prepared after standard cell culture.

Table S1: Additional data for the Oxford reference cohort.

Table S2: Additional data for the Oxford anemia clinic cohort.

Table S3-S4: Additional data for the neonatal cohort.

Table S5: Additional data for the Bangladeshi cohort.

Table S6: Additional data for the Peruvian cohort.

SUPPLEMENTARY METHODS

Measurements and information recorded for the Bangladeshi cohort: Adult and children included complicated and uncomplicated malaria patients admitted to hospital in Bangladesh with microscopy-confirmed *P. falciparum* infection. Healthy controls with no known acute or chronic illness (including hypertension or diabetes) were recruited at Chattogram Medical College Hospital. Antimalarial treatment was with intravenous artesunate (Guilin Pharmaceuticals, China), and/or artemether/lumefantrine (Coartem, Novartis, Switzerland). Supportive management was according to World Health Organization guidelines (Severe malaria. *Trop Med Int Health* **19 Suppl 1**, 7-131 (2014)) including a Foley catheter for urine output monitoring in comatose patients. On enrolment, clinical history and examination were performed and venous blood sample and urine sample collected by the clinical research physicians. All malaria smears and sample processing were performed on-site by clinical malaria laboratory technicians. Venous complete blood count was performed in Chattogram the same day of collection using a Sysmex TX-2000i (Sysmex Corporation, Kobe, Japan). Venous biochemistry was performed using point-of-care iSTAT analyser (Abbott Laboratories, USA); creatinine, LDH and bilirubin quantification were performed in Bangkok, Thailand (Olympus AU400 chemistry analyzer). Cell-free Hb was quantified from twice-centrifuged citrated plasma in Darwin, Australia (enzyme-linked immunosorbant assay; Bethyl Laboratories). Urine samples were assessed within one hour of collection for urinalysis using Combur-Test strip (Roche Diagnostics) and urine microscopy under high-power field of urine sediment after centrifugation for 5 min at 2000x. Urine, serum and plasma samples were stored in liquid nitrogen before transport to Bangkok where samples were stored at –80C at Mahidol-Oxford Tropical Medicine Research Unit until shipment to Oxford for urine CA1 quantification, Bangkok for biochemistry quantification, and Darwin for cell-free hemoglobin quantification.

Measurements and information recorded for the Peruvian cohort: Participants were tested on-site for malaria infection by standard microscopy protocols outlined in the Peruvian national guidelines ("Norma Técnica de Salud Para el Control de Calidad del Diagnóstico Microscópico de Malaria"). Briefly, two thick and thin blood smears were prepared from a finger-prick sample, stained with 10% Giemsa, and examined under a microscope with a 100x oil immersion objective. A second reading was performed at a reference center in Iquitos by a qualified microscopist supervised by the Laboratory of Public Health at the Peruvian National Institute of Health (INS). Participants completed a health-

related questionnaire relating to fever, illness symptoms in the past month, malaria infection history, malaria infection in the past month, and the use of antimalarial drugs during the past month. Blood Hb concentration was determined on-site, in duplicate, from a fingertip capillary blood sample using an HemoCue Hb 301 haemoglobinometer. Each participant provided blood collected from the antecubital vein of the non-dominant arm in 9-ml clot-activator tubes. Each participant also provided urine into a 100 mL sterile tube. Samples were anonymized and labelled with a participant identifier (site code and number), date of collection, and kept refrigerated (2-4°C). Urine and blood samples were accommodated in specially conditioned coolers for transport to the "UPCH-YALE Teodore E. Gildred" laboratories in the city of Iquitos (90 min travel time) for processing and storage. There, blood samples were centrifuged for 10 min at 2000g. Serum was transferred into 3 mL cryovials and stored at -80°C together with urine samples, until their transport on dry ice to UPCH in Lima for -80°C storage. Serum samples were analyzed in batches by Synlab clinical laboratories in Lima. Tests included immunoturbidimetry for Hp (mg/dL), ultra-sensitive assay for C-reactive protein (CRP; mg/dL), and by the diazotization reaction for total and conjugated bilirubin analyzed spectrophotometrically. Unconjugated bilirubin was obtained from the difference. Urine samples were transported in one batch under cold storage to Oxford for measurements.

Measurements and information recorded for the London cohort: Relevant clinical information was collected as part of clinical care, including serum and transcutaneous bilirubin, blood hemoglobin and full blood count, CRP and renal function, gestational age, birth weight, sex, result of direct antiglobulin test (DAT), reason for admission. Urine was collected using neonatal urine bags or cotton balls placed in nappies if bag collection was problematic. Urine was then transferred into 1.5 mL screw cap tubes containing 15 µL of Halt Protease Inhibitor Cocktail (Life Technologies, 87786) and immediately frozen at -20°C. Sample volumes ranged from 250 µL to 1 mL. Each tube was anonymized and uniquely labelled with a patient identifier and postnatal day at the point of collection. Samples were transported in four batches on dry ice, with temperature monitoring, to Oxford for measurements.

Measurements and information recorded for the Oxford cohort: Urine samples were self-collected by volunteers into 50 mL Falcon tubes and labeled with age and sex, and immediately placed into polystyrene containers with cooling blocks and maintained at low temperature until twice-daily collection. Upon retrieval, each tube was assigned an alphanumeric identifier, supplemented with protease inhibitor (cOmplete™, Roche/Merck),

and stored at –20°C for short-term preservation. CA1 analysis was performed at the earliest opportunity. No additional demographic or clinical data were collected, and all samples were destroyed immediately following analysis.

Urine measurement strategy and calibration: Measurements were performed in batches, each including a standardized positive control. Blood-based controls were prepared by diluting human finger-prick blood in nominally CA1-negative urine at dilution factors of 1:10,000, 1:100,000, and 1:1,000,000. CA1 standards were obtained by spiking recombinant human CA1 (rhCA1, Novus, 2180-CA-050) into nominally CA1-negative urine to obtain final concentrations of 50ng/100µL, 10 ng/100µL, 2 ng/100µL and 0.04ng/100µL. Larger urine volumes collected from adult donors enabled technical replicates.

Carbonic Anhydrase 1 sandwich ELISA: A Nunc Maxisorp 96-well plate was pre-coated with 0.25 µg/100 µL per well of capture goat anti-hCA1 polyclonal antibody (Novus/Abnova, NB120-6619-0.1mg), diluted in PBS, and incubated overnight at 4°C. The following day, the plate was blocked with 200 µL of SuperBlock T20 (PBS) Blocking Buffer (Life Technologies) for 90-120 min at room temperature (RT) on a shaker set to 550 rpm. The plate was rinsed four times with 0.05% Tween-20 in PBS (PBST). Next, 100 µL of urine was added per well, performed in triplicate, and incubated for 120 min at RT on a shaker (550 rpm). After incubation, the plate was rinsed four times with PBST. Subsequently, 100 µL/well of biotinylated (Biotinylation kit type A, Abcam, ab201795) detector mouse monoclonal antibody α-hCAI-7G12 (Novus/Abnova, H00000759-M07), diluted 525x in SuperBlock T20 (PBS), was added and incubated for 120 min at RT on a shaker (550 rpm). Following another rinse with PBST performed four times, the plate was incubated for 60 min with poly-streptavidin HRP (Life Technologies, 21140) diluted 2000x in SuperBlock T20 (PBS). After a final four-time rinse in PBST, the plate was developed using 3,3',5,5'-Tetramethylbenzidine Liquid Substrate, Supersensitive, for ELISA (T4444, Merck), and the reaction was stopped with Stop Reagent for TMB Substrate (Merck, 450nm, S5814). Absorbance was measured at 450 nm and corrected for pathlength measured at 570 nm using a Biotek Cytation 5 reader.

Indirect Hbα ELISA: A Nunc Maxisorp 96-well plate was incubated with 50 µL/well of urine overnight at 37°C. The following day, the plate was rinsed three times with 0.05% Tween-20 on PBS (PBST) and blocked with 200 µL of SuperBlock T20 (PBS) Blocking Buffer (Life Technologies) for 90-120 min at RT on a shaker set to 550 rpm. Next, 50 µL/well of anti-Hemoglobin Alpha Polyclonal rabbit antibody (Proteintech, 14537-1-AP), diluted 1:1000 in SuperBlock T20 (PBS), was added and incubated for 120 min at RT on a shaker (550 rpm).

Following this, the plate was rinsed four times with PBST and incubated with 50 µL/well of goat anti-Rabbit IgG (H+L) Poly-HRP Secondary Antibody (Life Technologies, 32260), diluted 1:5000 in SuperBlock-PBST, for 60 minutes at RT. After the final four-time rinse with PBST, the plate was developed using 3,3',5,5'-Tetramethylbenzidine Liquid Substrate, Supersensitive, for ELISA (Merck, T4444), and the reaction was stopped with Stop Reagent for TMB Substrate (Merck, S5814). Absorbance was measured at 450 nm and corrected for pathlength measured at 570 nm using a Biotek Cytation 5 reader.

Immunoblotting for human CA1, albumin and Hbα: Samples were prepared by mixing 21 µL of urine with 9 µL of reducing 4x Laemmli buffer (Bio-Rad), then loaded into a 10% polyacrylamide gel, separated, and transferred onto a PVDF membrane using a rapid blot system (Bio-Rad). Membranes were blocked in 5% skimmed milk prepared in 0.1% PBST for 1 h at RT. After blocking, membranes were cut between the 37 kDa and 50 kDa marks. The top membrane was incubated overnight at 4°C with mouse monoclonal anti-albumin antibody (1:5,000, Proteintech, 66051-1), and the lower membrane was incubated with rabbit recombinant monoclonal anti-human CA1 (EPR5192) antibody (1:1000, Abcam, ab124976). The next day, membranes were washed in 0.1% PBST and incubated with poly HRP-conjugated secondary antibodies diluted 1:10,000 in 5% skimmed milk in 0.1% PBST for 1 hour at RT (Goat anti-Rabbit IgG (H+L) Poly-HRP (32260) and Goat anti-Mouse IgG (H+L) Poly-HRP (32230), Life Technologies). After a final wash in 0.1% PBST, membranes were developed using SuperSignal West Dura Extended Duration Substrate and visualized using a ChemiDoc system (Bio-Rad). The bottom membrane was then stripped and redeveloped overnight at 4°C using 1:2,000 diluted anti-Hemoglobin Alpha Polyclonal rabbit primary antibody (Proteintech, 14537-1-AP) and secondary Goat anti-Rabbit IgG (H+L) Poly-HRP (1:10,000, Life Technologies, 32260).

Lateral flow device development and T/C line densitometry: A nitrocellulose membrane (Sartorius, 1UN14ER100025NT) was coated with a rabbit monoclonal anti-carbonic anhydrase 1 (CA1) antibody (Abcam, EPR23232-14) to form the test (T) line, and with an anti-rabbit IgG antibody (Abcam, AX32) to form the control (C) line. The membrane was integrated into a lateral flow strip by attaching a sample pad and conjugate pad (Merck) to one end (proximal to the T line), and an absorbent pad (Merck) to the opposite end (proximal to the C line). The conjugate pad was pre-treated with 40 nm colloidal gold (Merck)–labeled rabbit monoclonal anti-CA1 detector antibody (Abcam, EPR23232-66). The completed strip was enclosed within a plastic cassette (GeneSign Biotech) featuring a sample

well and a viewing window for visualizing T and C line development. For each test, 100 μL of undiluted urine was applied to the sample well. After a 10-minute incubation at room temperature, results were recorded. T and C lines were quantified densitometrically using Fiji software as area under the curve after background offset.

Assessment of Antibody Cross-Reactivity: To evaluate potential cross-reactivity of antibodies used in the sandwich ELISA and lateral flow device (LFD) with carbonic anhydrase 2 (CA2), the isoform most homologous to CA1, recombinant human CA2 (rhCA2; Novus, 2184-CA-050) was diluted in nominally CA1-free urine to achieve final concentrations ranging from 0.04 to 100 ng per 100 μL. To generate a protein-rich matrix containing various carbonic anhydrase isoforms, including CA2 and the membrane-associated isoforms CA9 and CA12, HT29 colorectal cancer cells were lysed in RIPA buffer. The total protein concentration of the resulting lysate was determined using a bicinchoninic acid (BCA) assay (Pierce, Life Technologies) and subsequently diluted in CA1-negative urine to yield final total protein concentrations of 50, 25, and 20 μg per 100 μL.

Data processing and statistical analyses. Relevant clinical information collected at the London, Bangladesh, or Peru sites was transferred securely for analysis in Oxford as password-protected files, ensuring anonymity of samples. Data were carefully curated and appended with measurements performed on urine: ELISA (absorbance measurements) for CA1 and Hb, western blot (densitometry and its visual score) for CA1, Hb and albumin, and LFD T/C ratio for CA1 immunoreactivity. The datasets and analysis code (MATLAB) are included in supplemental information. After confirming normality (Kolmogorov-Smirnov test), statistical testing included Pearson's or Spearman's correlation and one-way ANOVA for comparing >2 groups, Fisher's exact test or Chi-squared for contingency tables. Principal component analysis used standardized data. Categorization strategy was informed by decision tree.

FIGURE S1

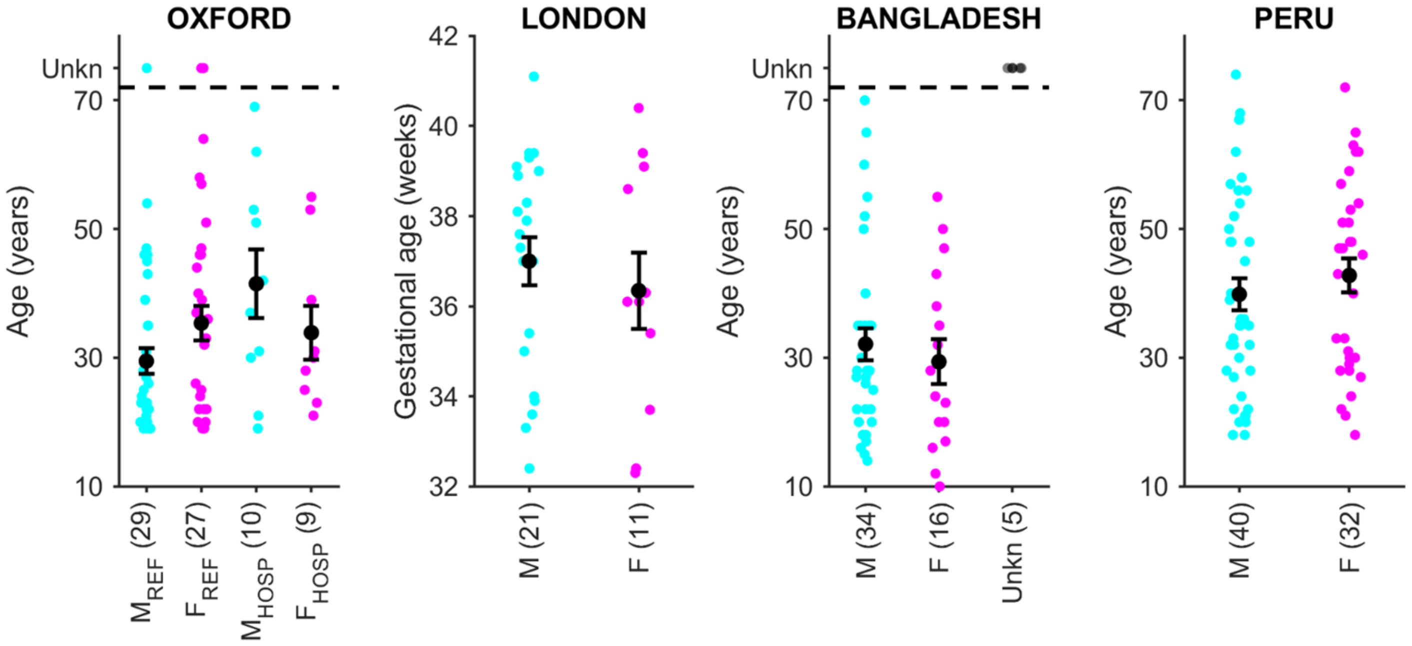

OXFORD
LONDON
BANGLADESH
PERU
Age (years)
Gestational age (weeks)
Unkn
70
50
30
10
42
40
38
36
34
32
M_REF (29)
F_REF (27)
M_HOSP (10)
F_HOSP (9)
M (21)
F (11)
M (34)
F (16)
Unkn (5)
M (40)
F (32)

FIGURE S2

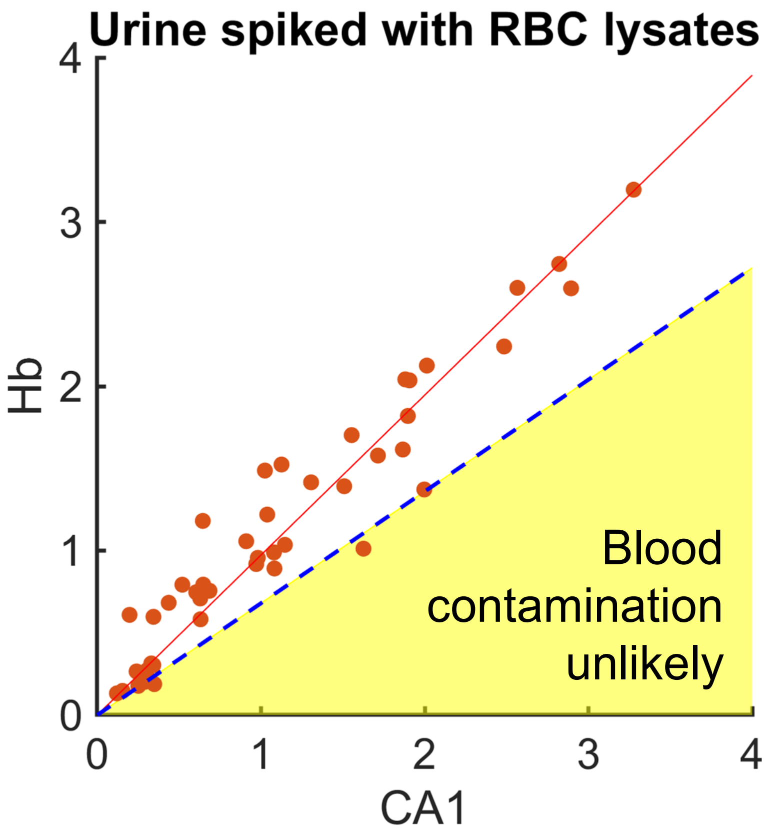
Urine spiked with RBC lysates
Hb
CA1
0
1
2
3
4
0
1
2
3
4
Blood
contamination
unlikely

FIGURE S3

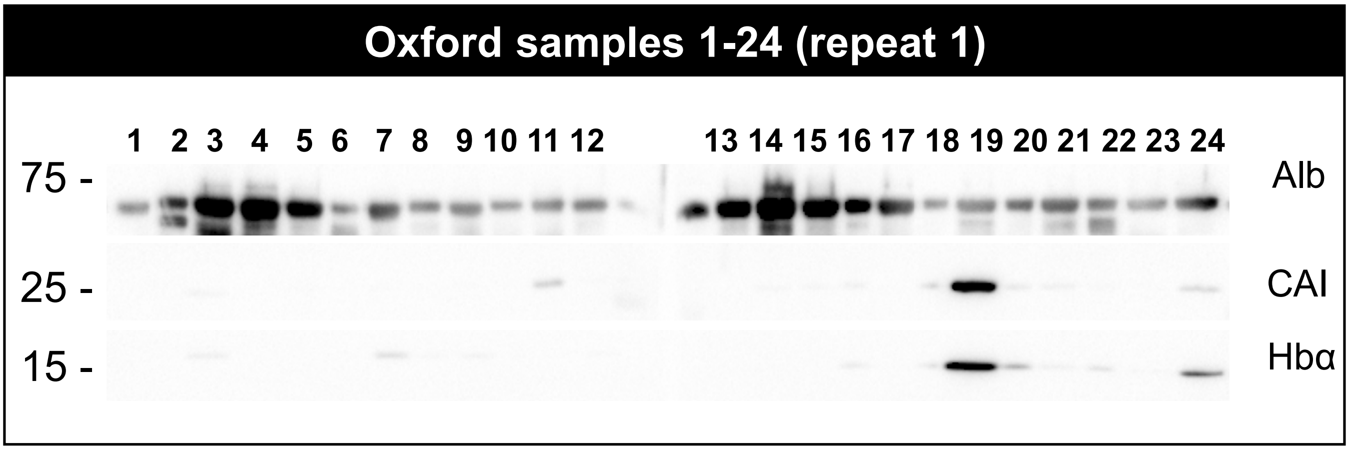
Oxford samples 1-24 (repeat 1)
1 2 3 4 5 6 7 8 9 10 11 12 13 14 15 16 17 18 19 20 21 22 23 24
75 -
25 -
15 -
Alb
CAI
Hbα

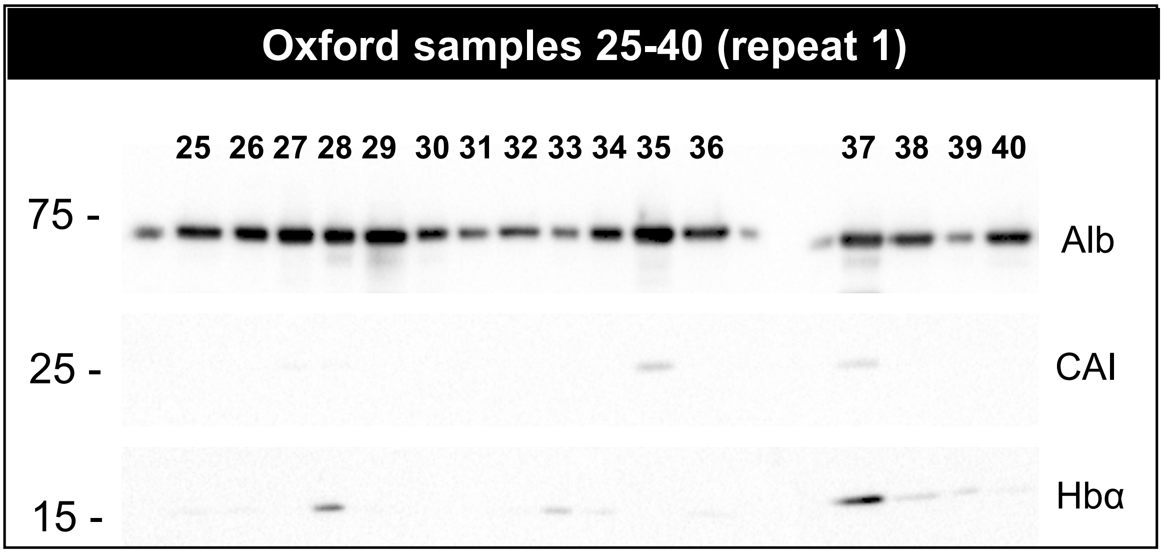
Oxford samples 25-40 (repeat 1)
25 26 27 28 29 30 31 32 33 34 35 36 37 38 39 40
75 -
25 -
15 -
Alb
CAI
Hbα

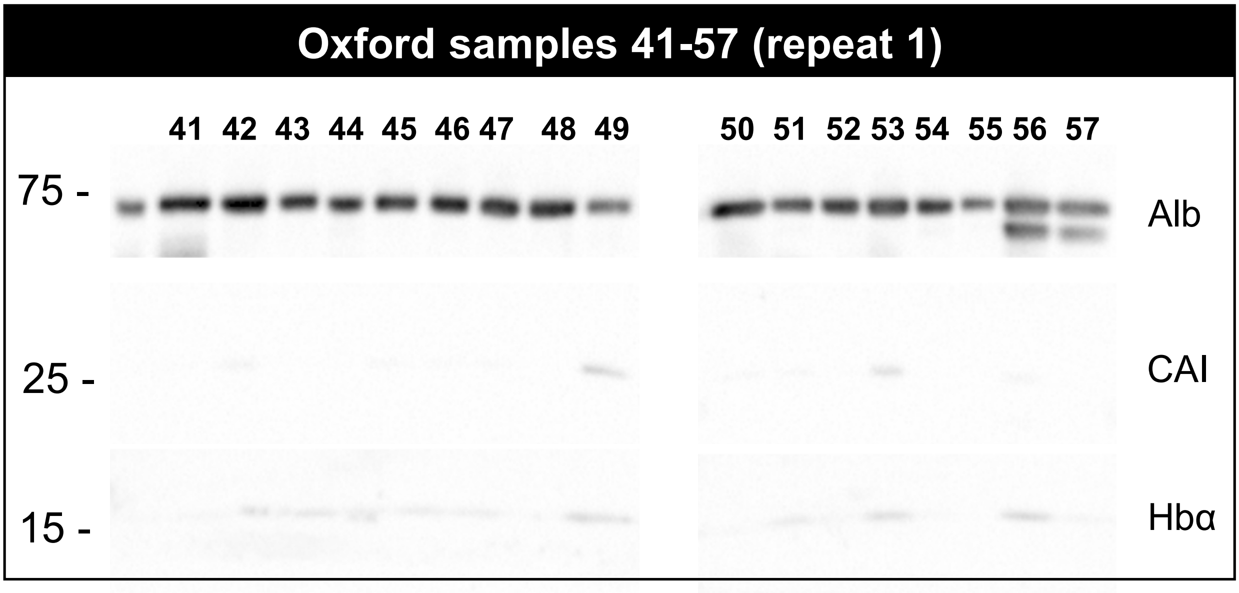
Oxford samples 41-57 (repeat 1)
41 42 43 44 45 46 47 48 49 50 51 52 53 54 55 56 57
75 -
25 -
15 -
Alb
CAI
Hbα

FIGURE S4

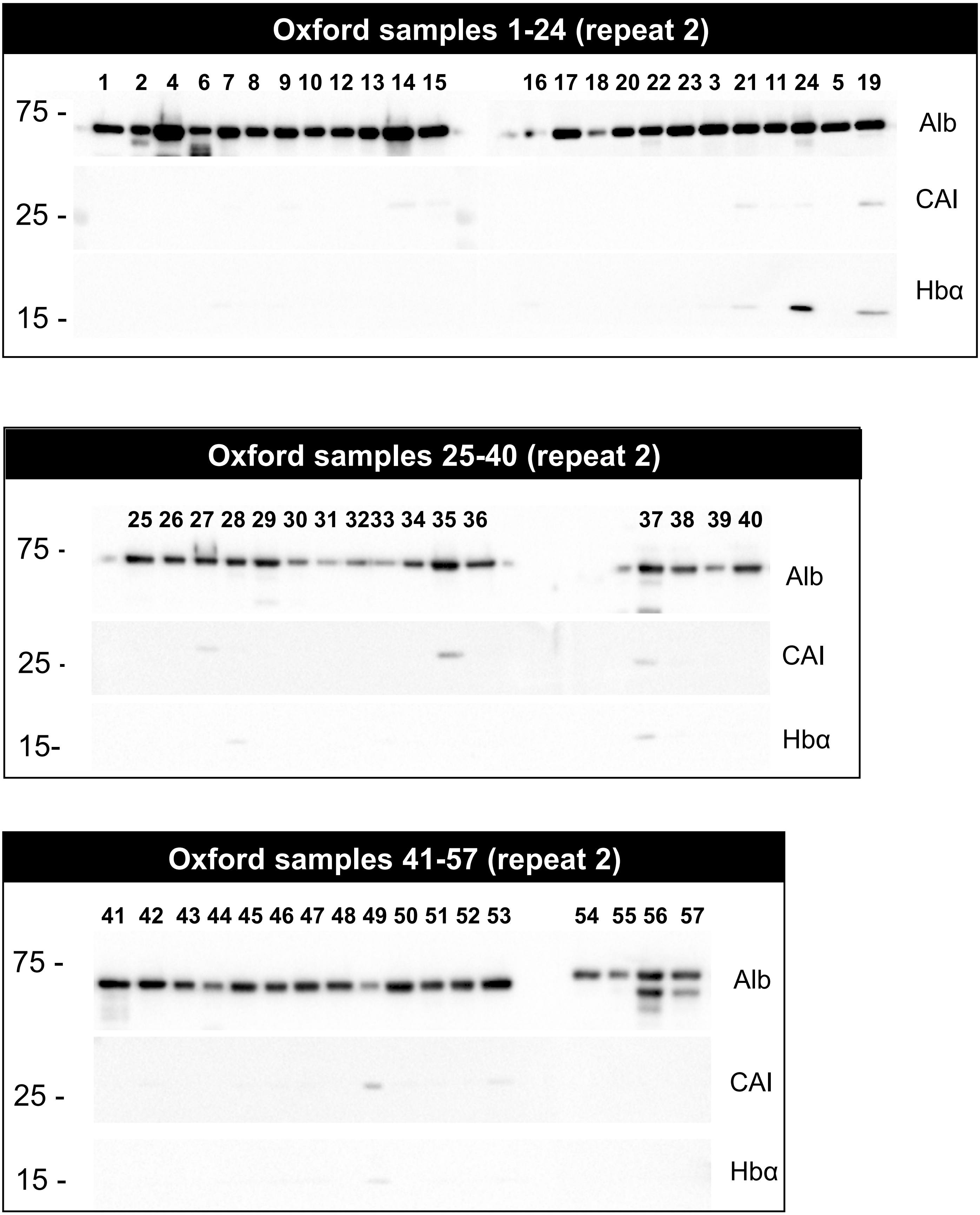
Oxford samples 1-24 (repeat 2)
1 2 4 6 7 8 9 10 12 13 14 15 16 17 18 20 22 23 3 21 11 24 5 19
75 -
25 -
15 -
Alb
CAI
Hbα
Oxford samples 25-40 (repeat 2)
25 26 27 28 29 30 31 3233 34 35 36 37 38 39 40
75 -
25 -
15-
Alb
CAI
Hbα
Oxford samples 41-57 (repeat 2)
41 42 43 44 45 46 47 48 49 50 51 52 53 54 55 56 57
75 -
25 -
15 -
Alb
CAI
Hbα

FIGURE S5

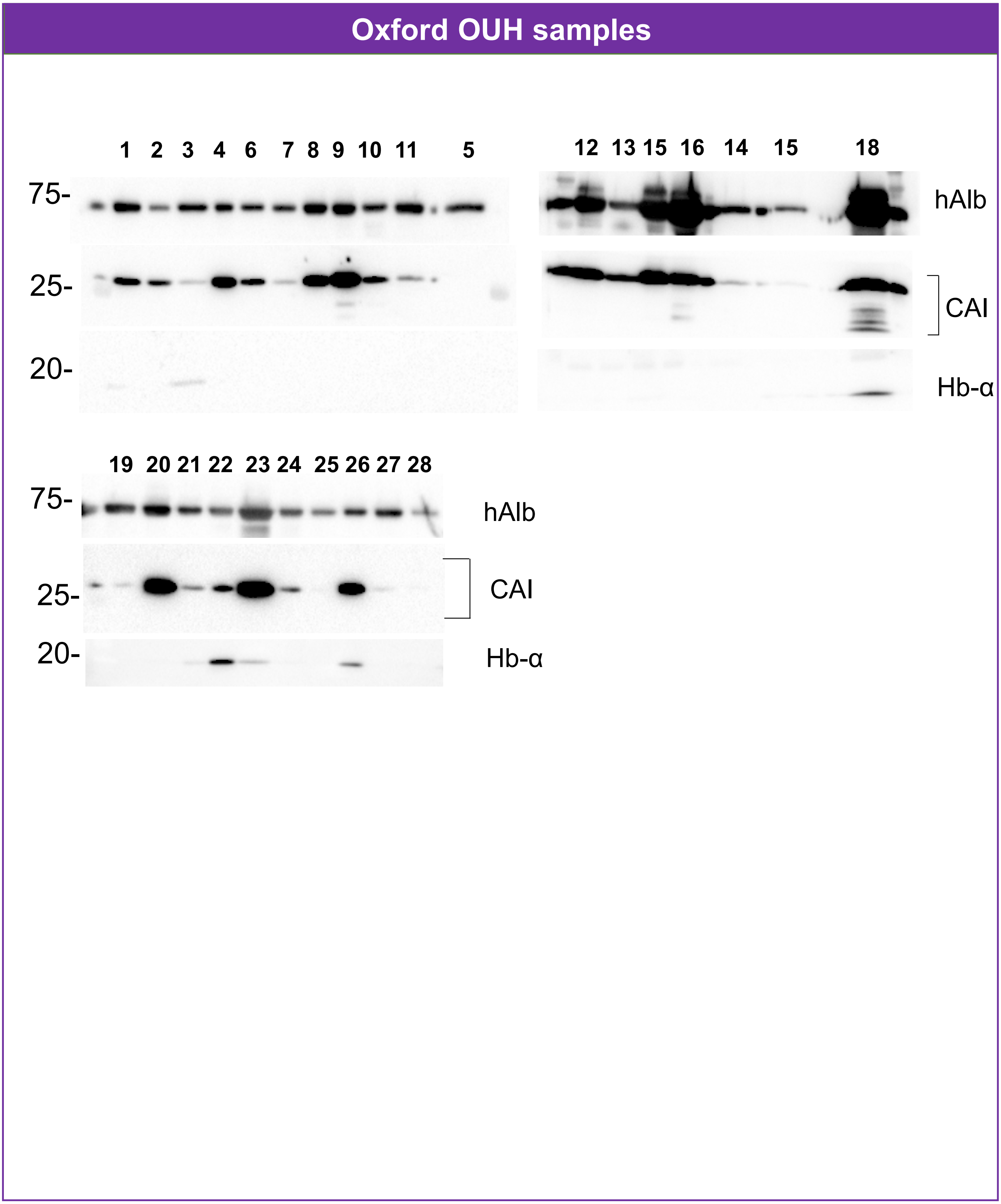

Oxford OUH samples
1 2 3 4 6 7 8 9 10 11 5
12 13 15 16 14 15 18
75-
25-
20-
hAlb
CAI
Hb-α
19 20 21 22 23 24 25 26 27 28
75-
25-
20-
hAlb
CAI
Hb-α

FIGURE S6

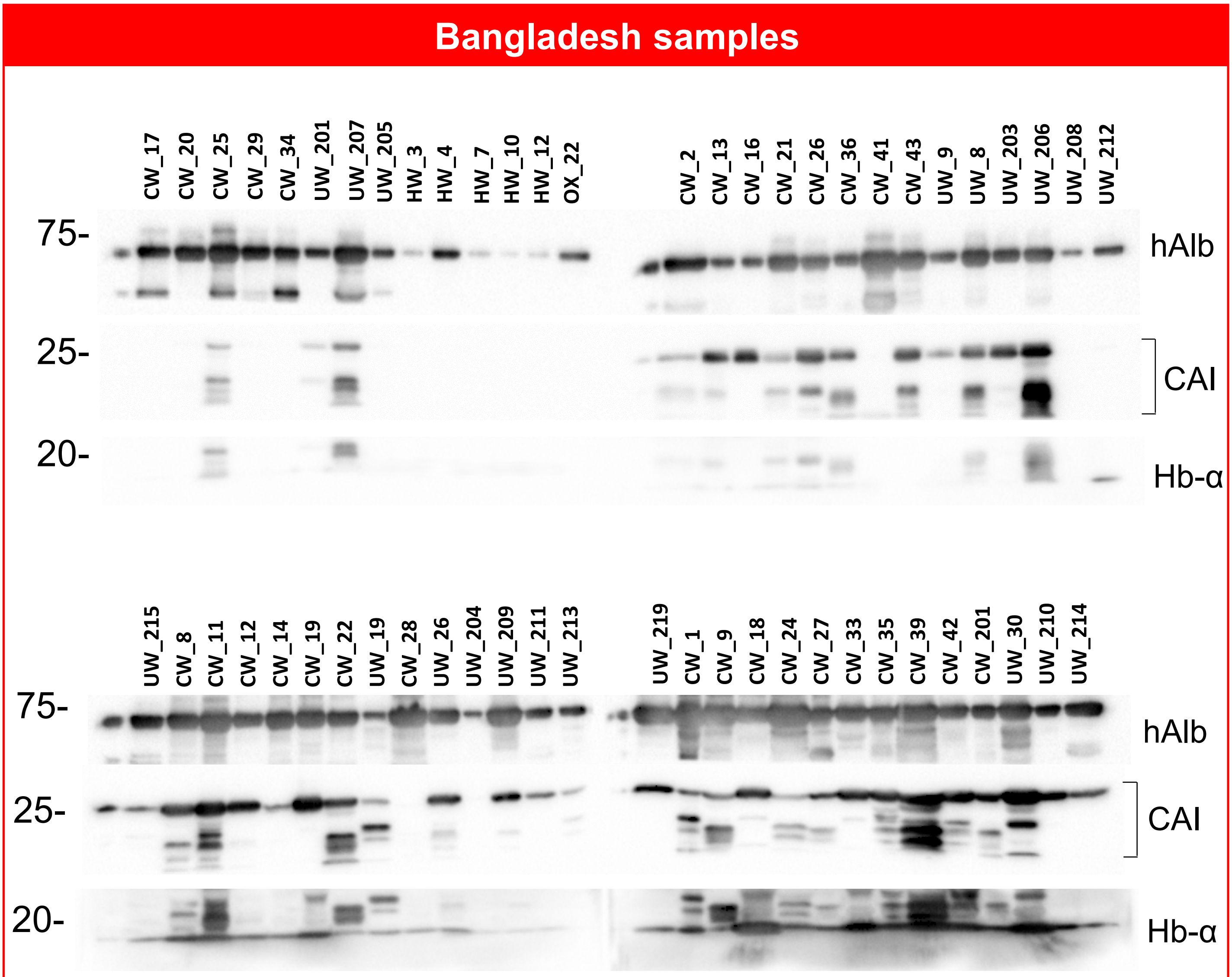

Bangladesh samples
CW_17
CW_20
CW_25
CW_29
CW_34
UW_201
UW_207
UW_205
HW_3
HW_4
HW_7
HW_10
HW_12
OX_22
CW_2
CW_13
CW_16
CW_21
CW_26
CW_36
CW_41
CW_43
UW_9
UW_8
UW_203
UW_206
UW_208
UW_212
75-
25-
20-
hAlb
CAI
Hb-α
UW_215
CW_8
CW_11
CW_12
CW_14
CW_19
CW_22
UW_19
CW_28
UW_26
UW_204
UW_209
UW_211
UW_213
UW_219
CW_1
CW_9
CW_18
CW_24
CW_27
CW_33
CW_35
CW_39
CW_42
CW_201
UW_30
UW_210
UW_214
75-
25-
20-
hAlb
CAI
Hb-α

FIGURE S7

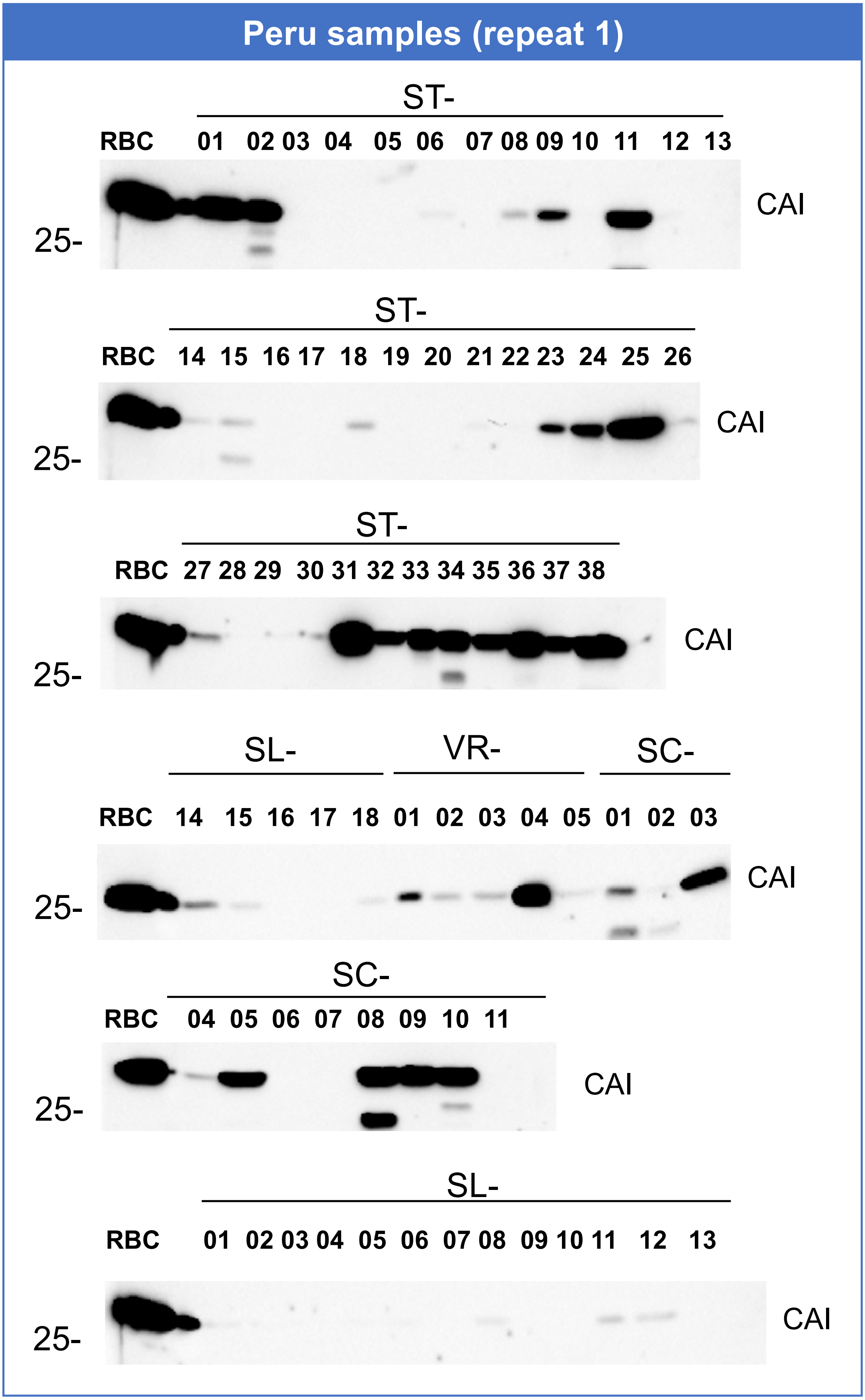
Peru samples (repeat 1)
ST-
RBC 01 02 03 04 05 06 07 08 09 10 11 12 13
25-
CAI
ST-
RBC 14 15 16 17 18 19 20 21 22 23 24 25 26
25-
CAI
ST-
RBC 27 28 29 30 31 32 33 34 35 36 37 38
25-
CAI
SL-
VR-
SC-
RBC 14 15 16 17 18 01 02 03 04 05 01 02 03
25-
CAI
SC-
RBC 04 05 06 07 08 09 10 11
25-
CAI
SL-
RBC 01 02 03 04 05 06 07 08 09 10 11 12 13
25-
CAI

FIGURE S8

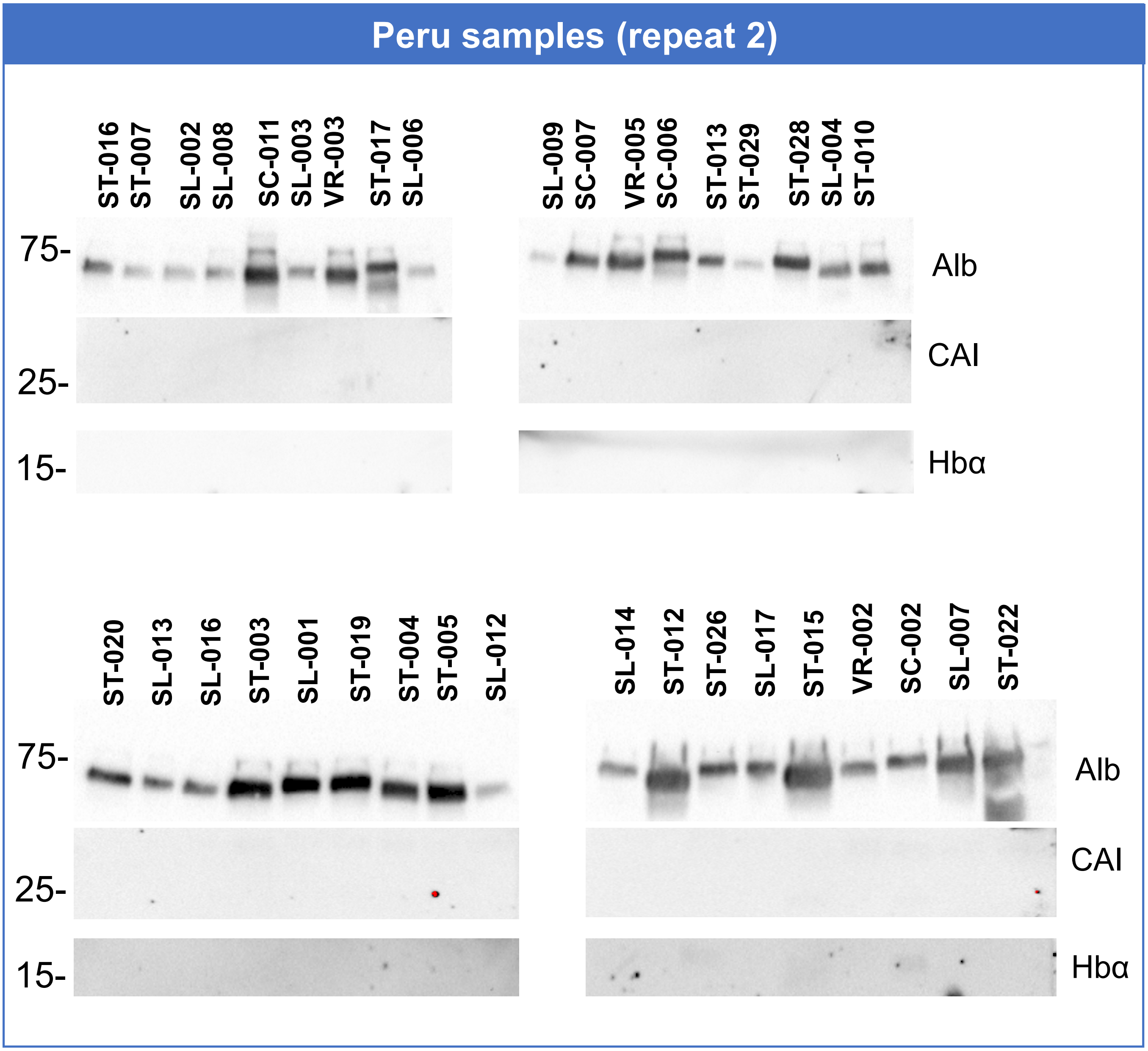
Peru samples (repeat 2)
ST-016
ST-007
SL-002
SL-008
SC-011
SL-003
VR-003
ST-017
SL-006
SL-009
SC-007
VR-005
SC-006
ST-013
ST-029
ST-028
SL-004
ST-010
75-
25-
15-
Alb
CAI
Hbα
ST-020
SL-013
SL-016
ST-003
SL-001
ST-019
ST-004
ST-005
SL-012
SL-014
ST-012
ST-026
SL-017
ST-015
VR-002
SC-002
SL-007
ST-022
75-
25-
15-
Alb
CAI
Hbα

FIGURE S9

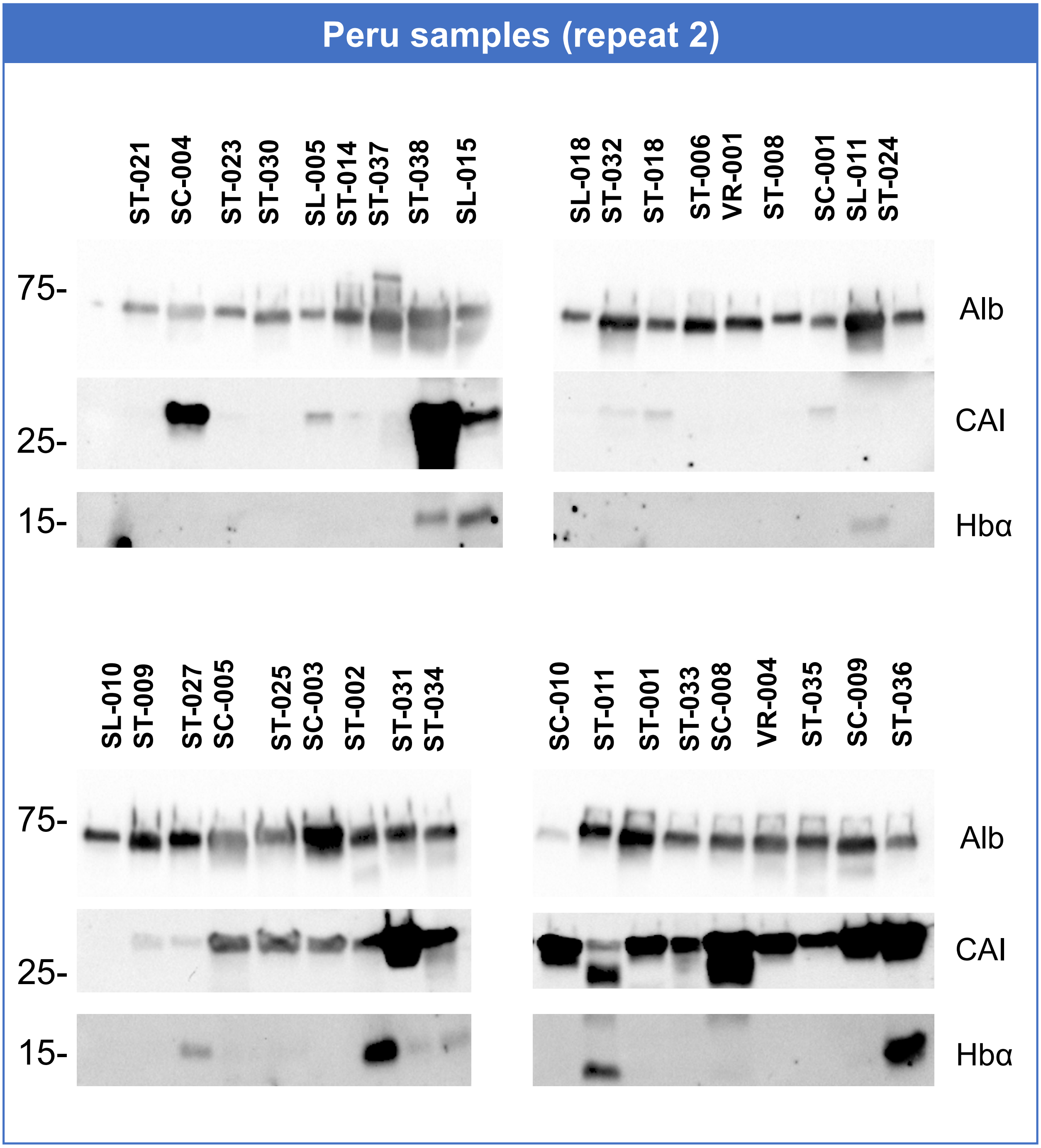
Peru samples (repeat 2)
ST-021
SC-004
ST-023
ST-030
SL-005
ST-014
ST-037
ST-038
SL-015
SL-018
ST-032
ST-018
ST-006
VR-001
ST-008
SC-001
SL-011
ST-024
75-
25-
15-
Alb
CAI
Hbα
SL-010
ST-009
ST-027
SC-005
ST-025
SC-003
ST-002
ST-031
ST-034
SC-010
ST-011
ST-001
ST-033
SC-008
VR-004
ST-035
SC-009
ST-036
75-
25-
15-
Alb
CAI
Hbα

FIGURE S10

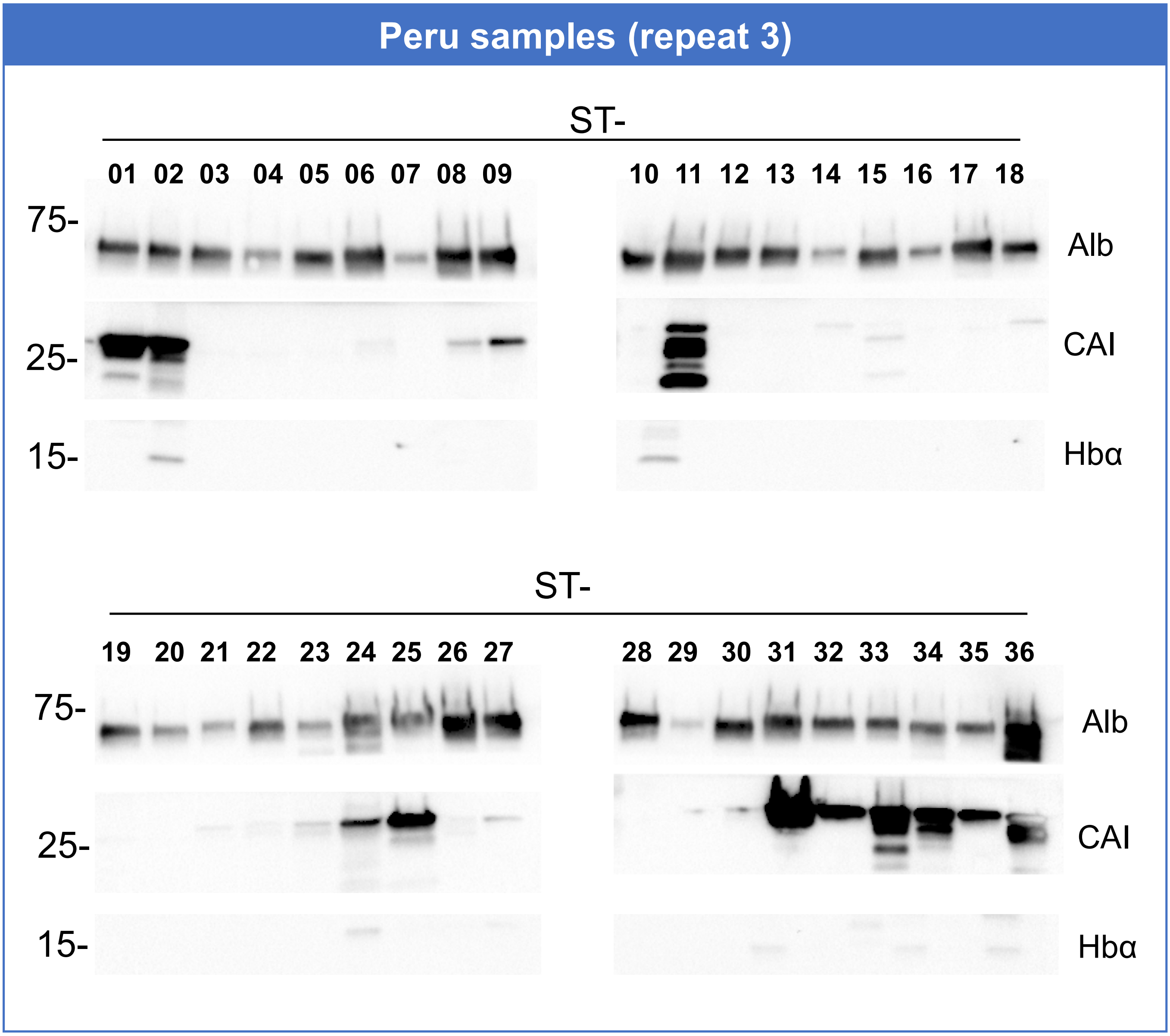
Peru samples (repeat 3)
ST-
01 02 03 04 05 06 07 08 09
10 11 12 13 14 15 16 17 18
75-
25-
15-
Alb
CAI
Hbα
ST-
19 20 21 22 23 24 25 26 27
28 29 30 31 32 33 34 35 36
75-
25-
15-
Alb
CAI
Hbα

FIGURE S11

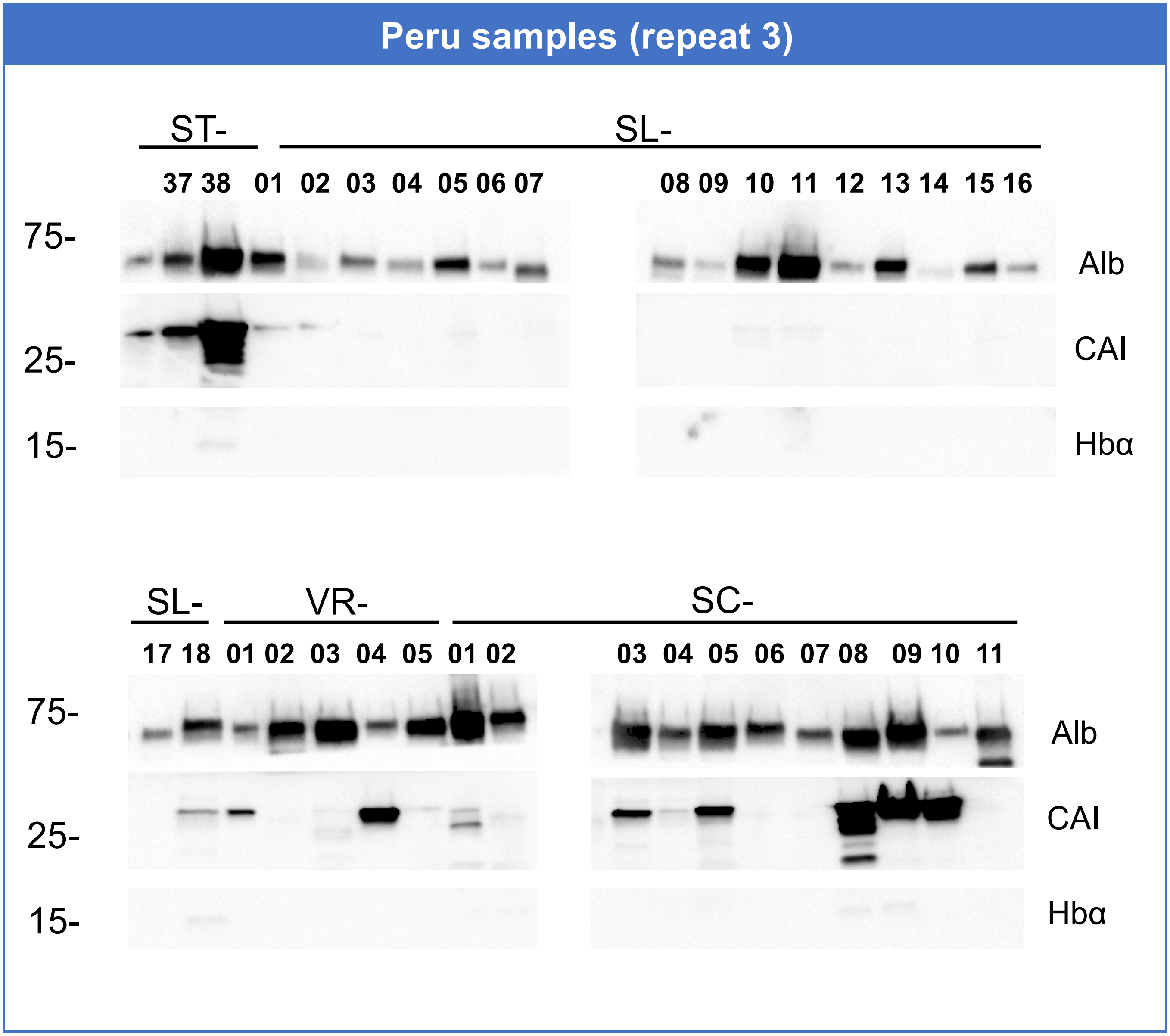

Peru samples (repeat 3)
ST-
SL-
37 38 01 02 03 04 05 06 07
08 09 10 11 12 13 14 15 16
75-
25-
15-
Alb
CAI
Hbα
SL-
VR-
SC-
17 18 01 02 03 04 05 01 02
03 04 05 06 07 08 09 10 11
75-
25-
15-
Alb
CAI
Hbα

FIGURE S12

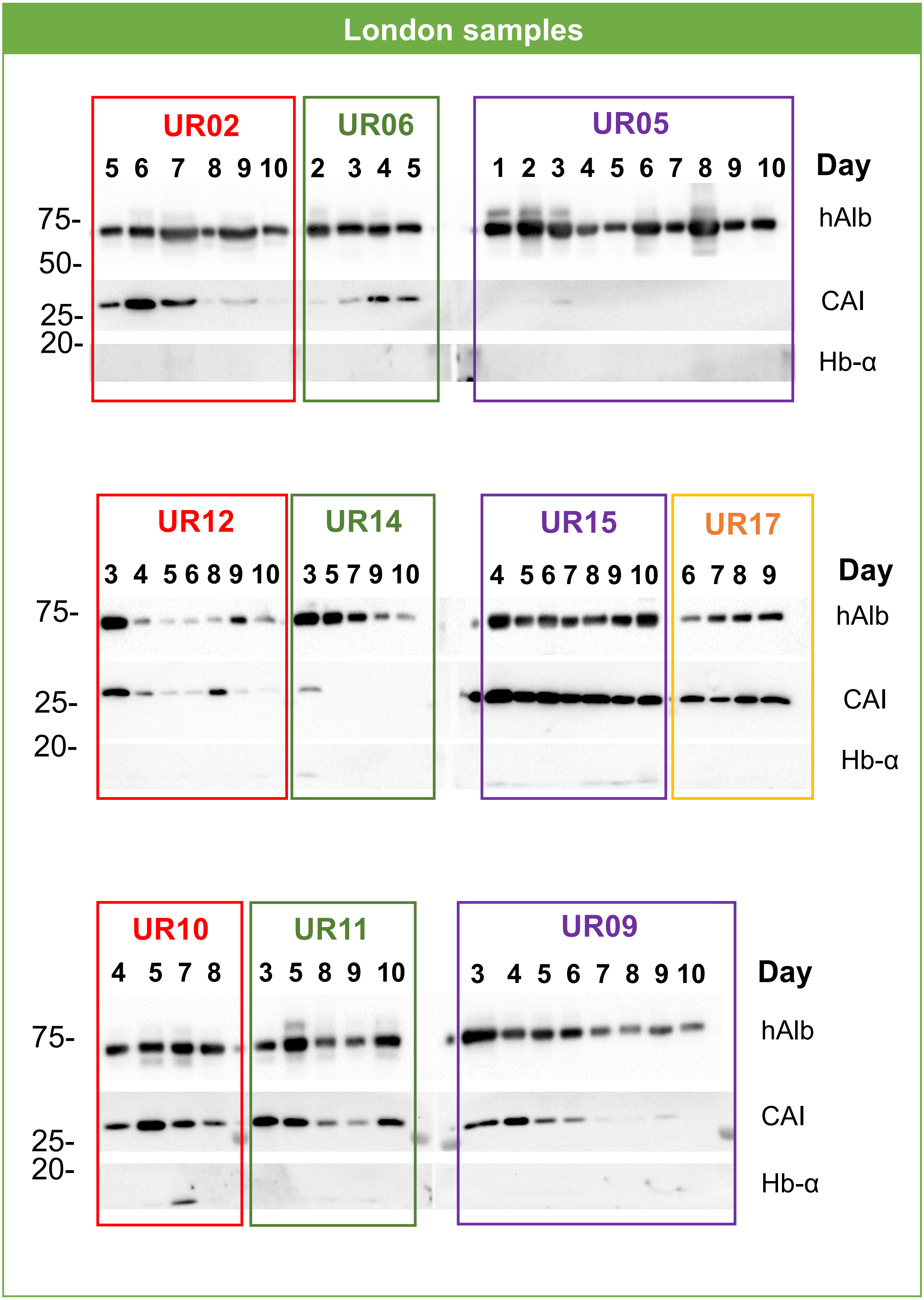
London samples
UR02
5 6 7 8 9 10
UR06
2 3 4 5
UR05
1 2 3 4 5 6 7 8 9 10
Day
75-
50-
25-
20-
hAlb
CAI
Hb-α
UR12
3 4 5 6 8 9 10
UR14
3 5 7 9 10
UR15
4 5 6 7 8 9 10
UR17
6 7 8 9
Day
75-
25-
20-
hAlb
CAI
Hb-α
UR10
4 5 7 8
UR11
3 5 8 9 10
UR09
3 4 5 6 7 8 9 10
Day
75-
25-
20-
hAlb
CAI
Hb-α

FIGURE S13

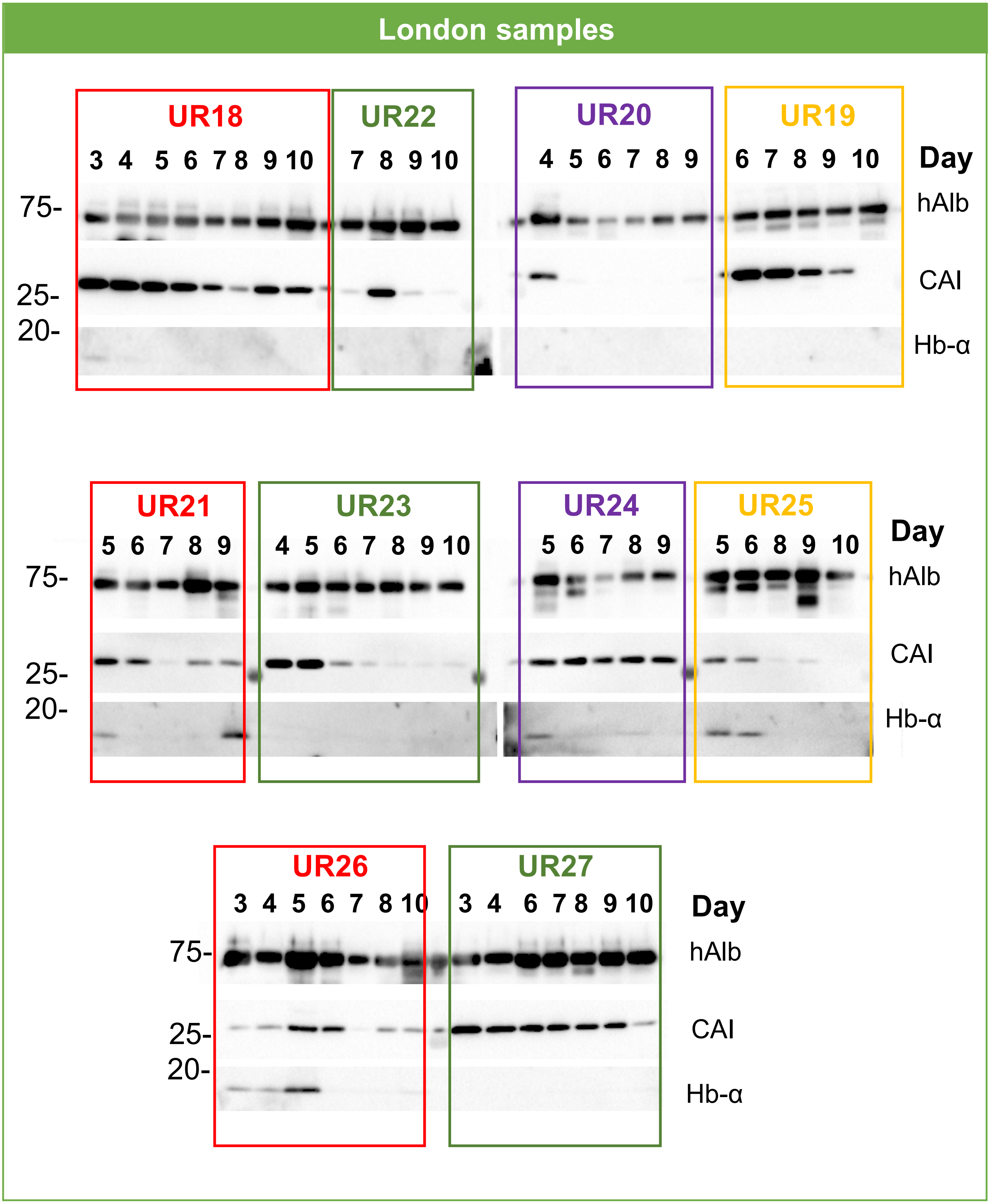
London samples
UR18
3 4 5 6 7 8 9 10
UR22
7 8 9 10
UR20
4 5 6 7 8 9
UR19
6 7 8 9 10
Day
75-
25-
20-
hAlb
CAI
Hb-α
UR21
5 6 7 8 9
UR23
4 5 6 7 8 9 10
UR24
5 6 7 8 9
UR25
5 6 8 9 10
Day
75-
25-
20-
hAlb
CAI
Hb-α
UR26
3 4 5 6 7 8 10
UR27
3 4 6 7 8 9 10
Day
75-
25-
20-
hAlb
CAI
Hb-α

FIGURE S14

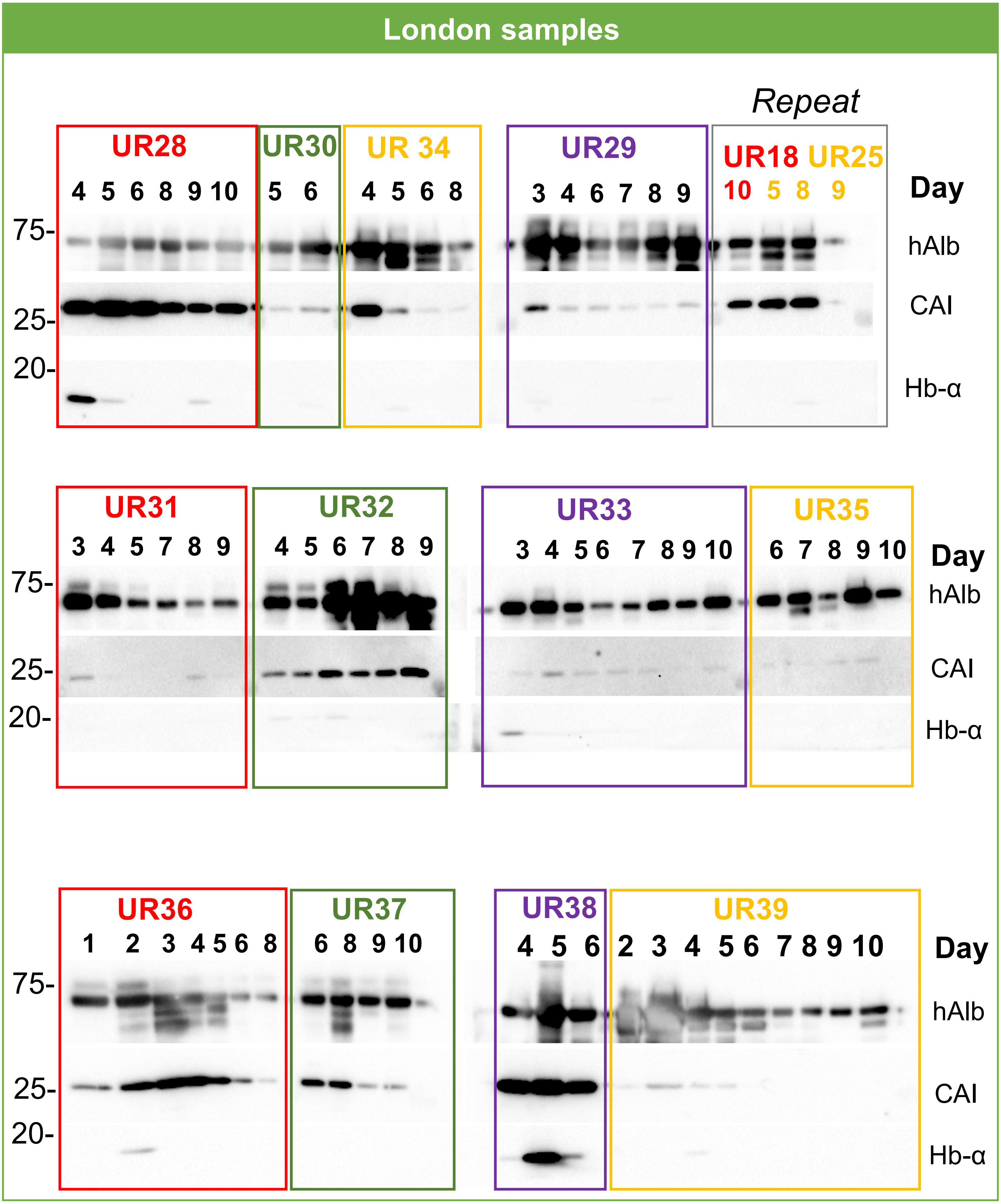
London samples
Repeat
UR28
4 5 6 8 9 10
UR30
5 6
UR 34
4 5 6 8
UR29
3 4 6 7 8 9
UR18 UR25
10 5 8 9
Day
75-
25-
20-
hAlb
CAI
Hb-α
UR31
3 4 5 7 8 9
UR32
4 5 6 7 8 9
UR33
3 4 5 6 7 8 9 10
UR35
6 7 8 9 10
Day
75-
25-
20-
hAlb
CAI
Hb-α
UR36
1 2 3 4 5 6 8
UR37
6 8 9 10
UR38
4 5 6
UR39
2 3 4 5 6 7 8 9 10
Day
75-
25-
20-
hAlb
CAI
Hb-α

FIGURE S15

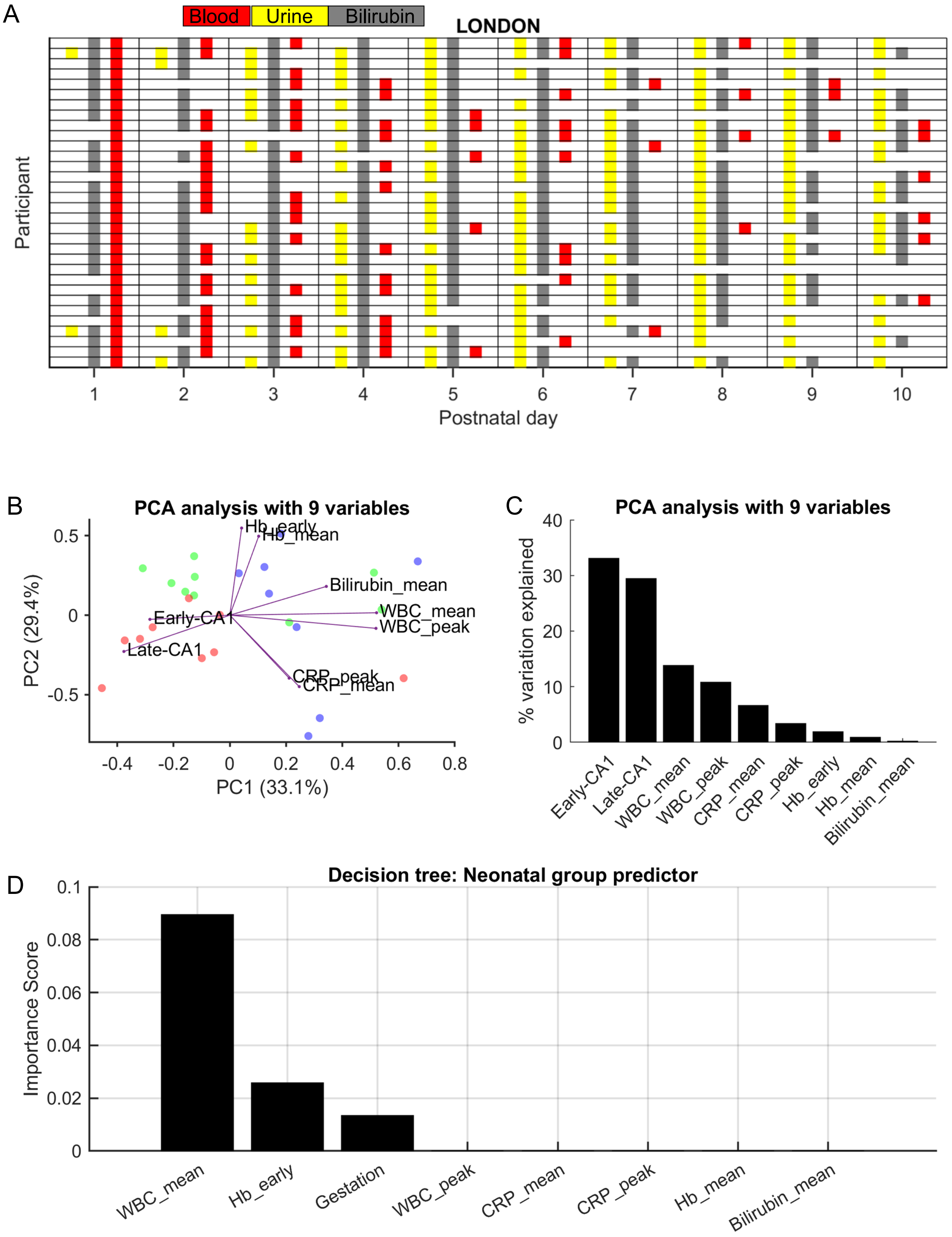
A
Blood
Urine
Bilirubin
LONDON
Participant
Postnatal day
1
2
3
4
5
6
7
8
9
10
B
PCA analysis with 9 variables
PC2 (29.4%)
PC1 (33.1%)
Hb_early
Hb_mean
Bilirubin_mean
WBC_mean
WBC_peak
Early-CA1
Late-CA1
CRP_peak
CRP_mean
C
PCA analysis with 9 variables
% variation explained
Early-CA1
Late-CA1
WBC_mean
WBC_peak
CRP_mean
CRP_peak
Hb_early
Hb_mean
Bilirubin_mean
D
Decision tree: Neonatal group predictor
Importance Score
WBC_mean
Hb_early
Gestation
WBC_peak
CRP_mean
CRP_peak
Hb_mean
Bilirubin_mean

FIGURE S16

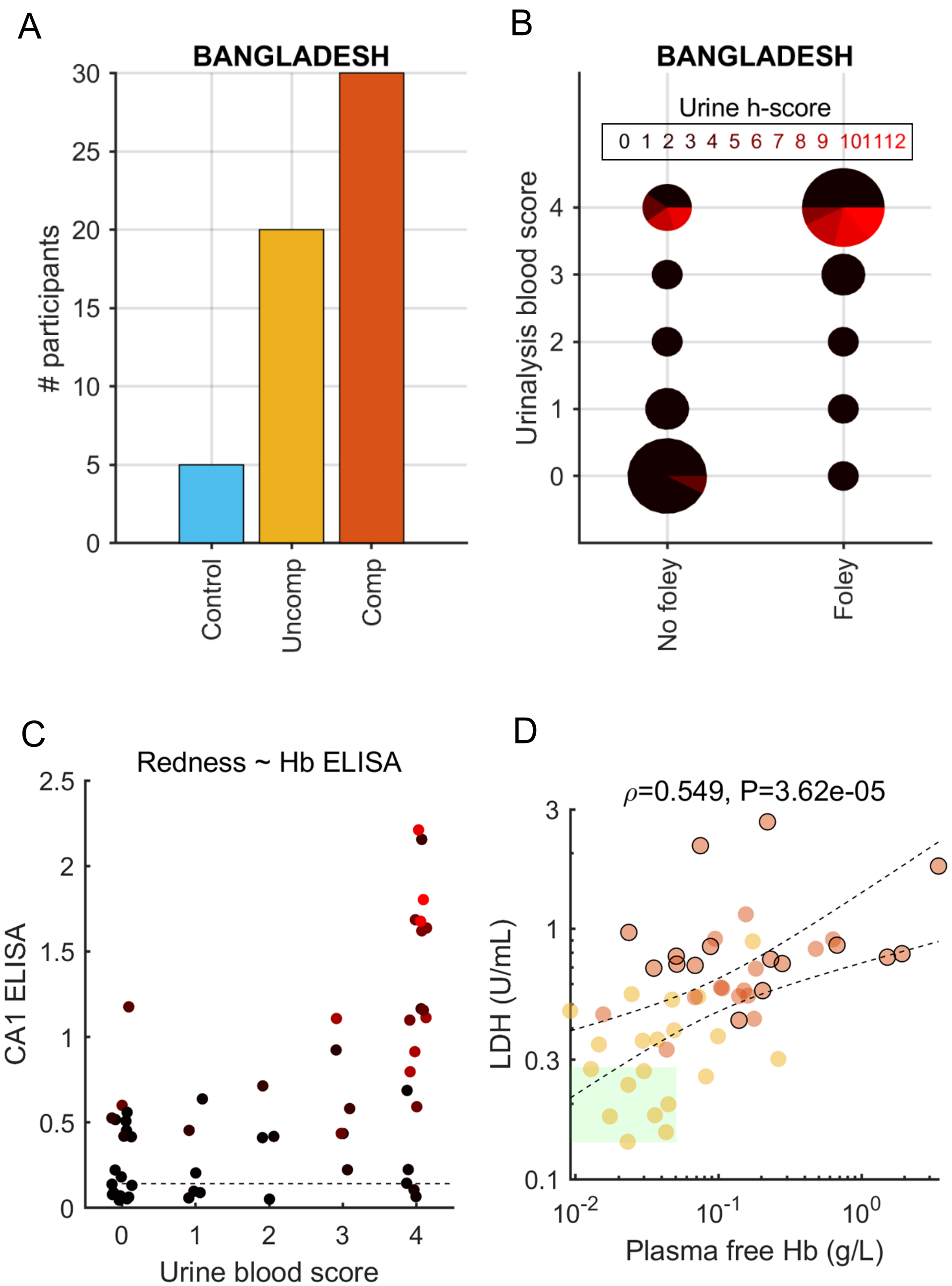
A
BANGLADESH
participants
30
25
20
15
10
5
0
Control
Uncomp
Comp
B
BANGLADESH
Urine h-score
0 1 2 3 4 5 6 7 8 9 101112
Urinalysis blood score
4
3
2
1
0
No foley
Foley
C
Redness ~ Hb ELISA
CA1 ELISA
2.5
2
1.5
1
0.5
0
0
1
2
3
4
Urine blood score
D
ρ=0.549, P=3.62e-05
LDH (U/mL)
3
1
0.3
0.1
10^{-2}
10^{-1}
10^{0}
Plasma free Hb (g/L)

FIGURE S17

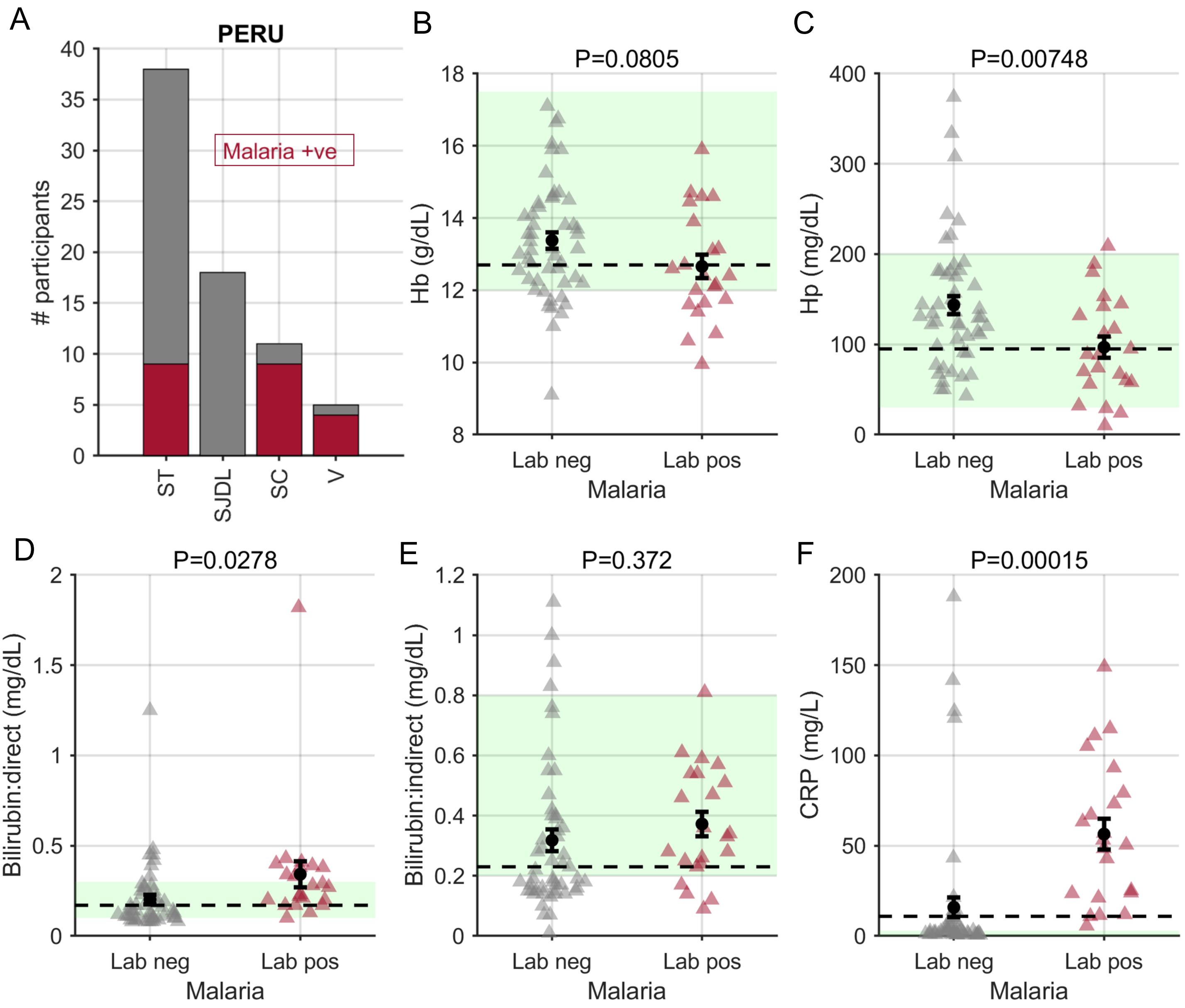
A
PERU
Malaria +ve
participants
ST
SJDL
SC
V
B
P=0.0805
Hb (g/dL)
Lab neg
Lab pos
Malaria
C
P=0.00748
Hp (mg/dL)
Lab neg
Lab pos
Malaria
D
P=0.0278
Bilirubin:direct (mg/dL)
Lab neg
Lab pos
Malaria
E
P=0.372
Bilirubin:indirect (mg/dL)
Lab neg
Lab pos
Malaria
F
P=0.00015
CRP (mg/L)
Lab neg
Lab pos
Malaria

FIGURE S18

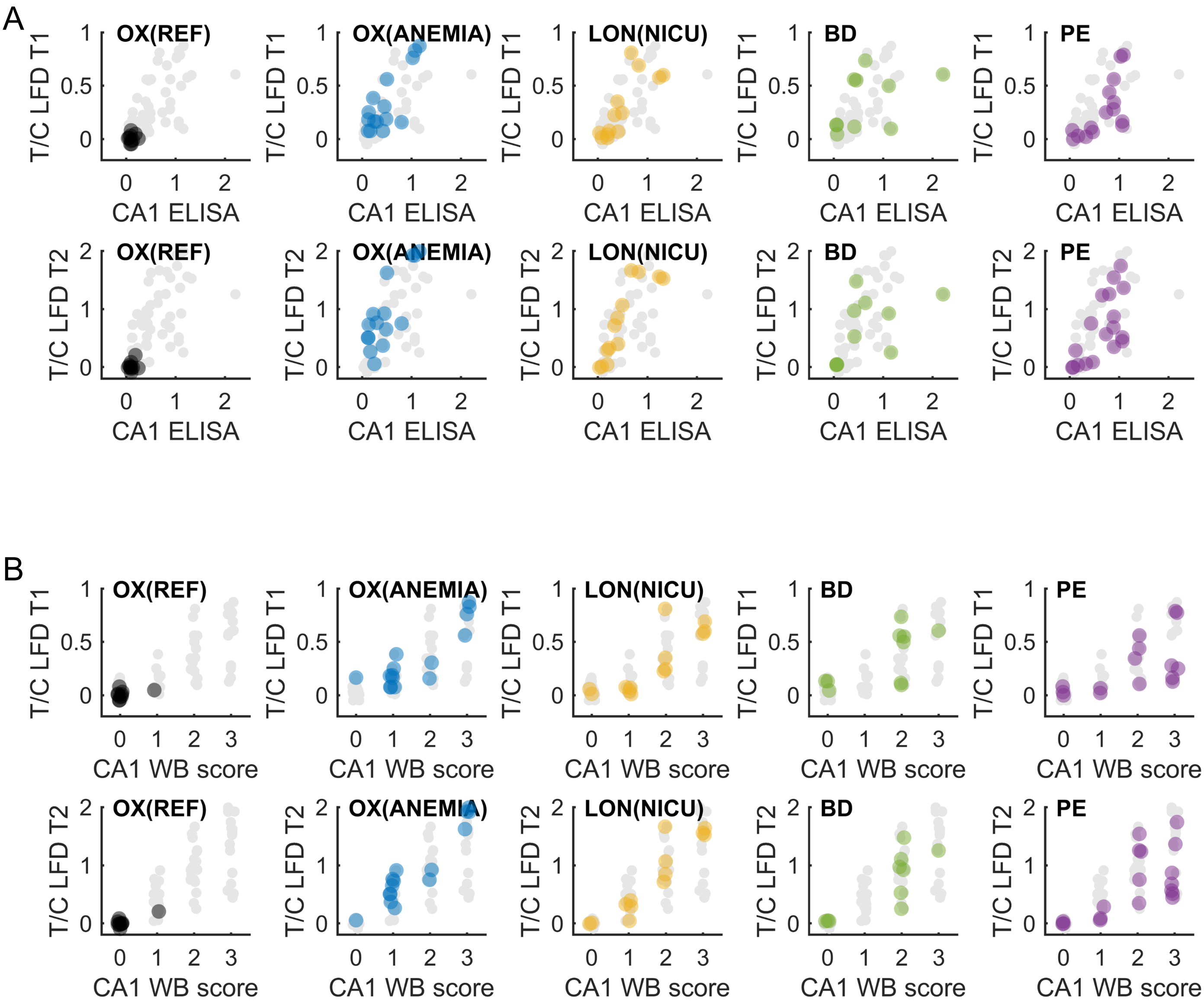
A
OX(REF)
OX(ANEMIA)
LON(NICU)
BD
PE
T/C LFD T1
T/C LFD T2
CA1 ELISA
B
OX(REF)
OX(ANEMIA)
LON(NICU)
BD
PE
T/C LFD T1
T/C LFD T2
CA1 WB score

FIGURE S19

A

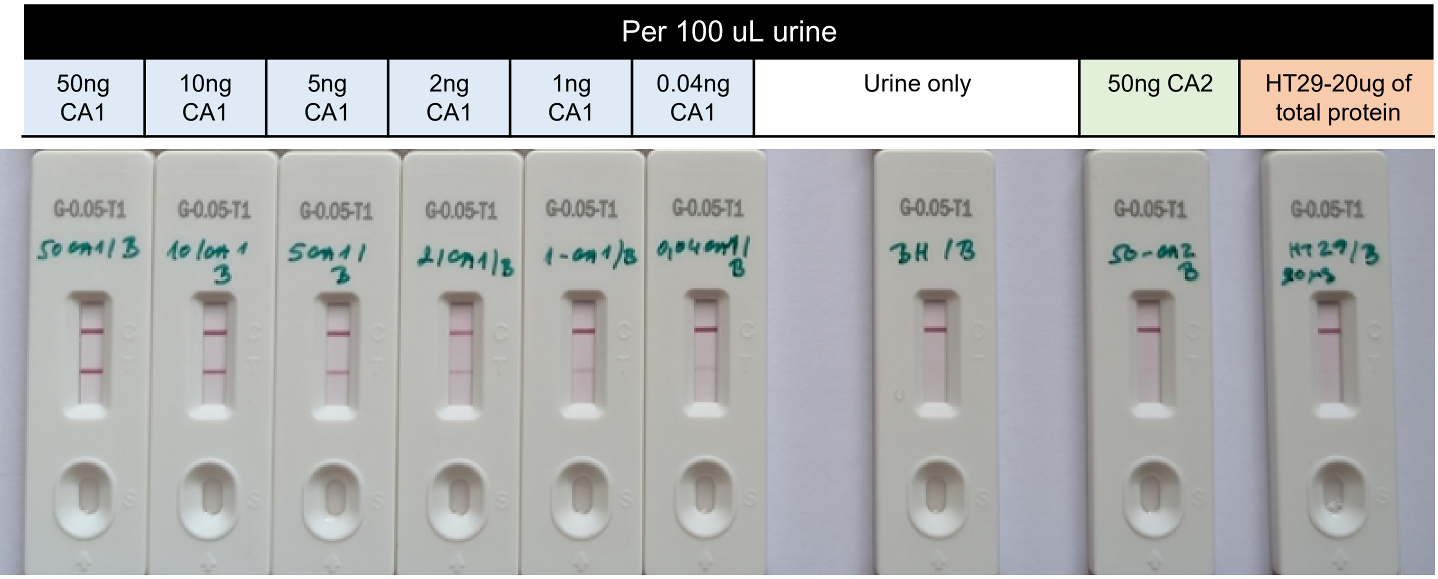

B

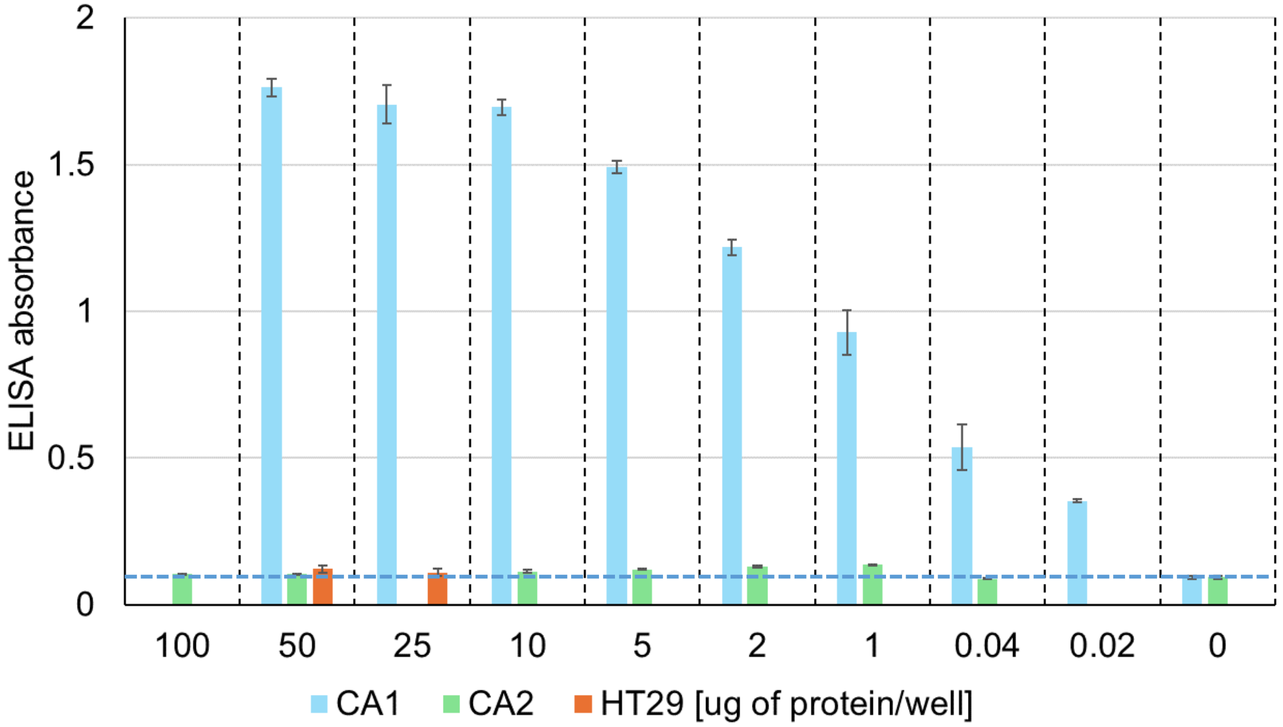

Supplement: Supplemental Methods and Figures [file BGLO_BGH-2025-000268-mmc1.pdf]
